# Emerging Nucleic Acid-Based Therapies for Hypercholesterolemia with Focus on a New Modality, Liver-Directed miR-30c Analog C2

**DOI:** 10.3390/cells15171575

**Published:** 2026-08-29

**Authors:** Rai Ajit K. Srivastava

**Affiliations:** 1 Mirnacures Therapeutics Inc., Boston, MA 02101-02117, USA; ajitsriva@gmail.com; Tel.: +1-215-801-5129; 2 NYU Grossman School of Medicine, NYU Langone (Long Island), Mineola, NY 11501, USA

**Keywords:** familial hypercholesterolemia, refractory hypercholesterolemia, nucleic acid therapeutics, siRNA, antisense oligonucleotide, gene therapy, genome editing, CRISPR, base editing, microRNA, miR-30c, MTTP, atherosclerosis

## Abstract

**Highlights:**

**Nucleic acid therapeutics for hypercholesterolemia**

- Antisense oligonucleotides and siRNA therapeutics have established clinically validated RNA-targeting strategies for LDL-C lowering, providing durable efficacy with infrequent dosing.
- Gene replacement and in vivo genome-editing technologies are advancing rapidly and hold promise for long-lasting, potentially one-time treatments for severe inherited dyslipidemias.
- microRNA-based therapeutics represent an emerging class of nucleic acid medicines that regulate networks of genes. The liver-directed miR-30c analog C2 has shown encouraging efficacy in lowering LDL-C and reducing atherosclerosis in animal models. However, further optimization is needed for clinical development.

**Abstract:**

Despite major advances in lipid-lowering therapies, a significant unmet need remains, particularly for patients with homozygous familial hypercholesterolemia (HoFH), severe heterozygous familial hypercholesterolemia (HeFH), and those who fail to achieve guideline-recommended LDL-C targets. Nucleic acid-based therapeutics have emerged as a transformative approach for treating hypercholesterolemia. Antisense oligonucleotides and small interfering RNAs (siRNAs) have demonstrated durable hepatic gene silencing and have led to approved therapies, while gene replacement and in vivo genome-editing strategies offer the potential for long-lasting, and possibly one-time, interventions. In parallel, microRNAs (miRNAs) have attracted increasing interest because of their ability to coordinately regulate multiple genes involved in lipoprotein metabolism, cholesterol transport, and lipid homeostasis. Human genetic studies further support the importance of miRNA-mediated regulation, exemplified by a rare ~2.5 kb deletion in the distal LDLR 3′UTR (“del2.5”) that disrupts miRNA-binding sites and is associated with lifelong low LDL-C levels. This review summarizes recent advances, mechanisms of action, clinical progress, and remaining challenges across antisense oligonucleotides, siRNAs, gene therapy, genome editing, and emerging miRNA-based therapeutics for hypercholesterolemia. As an example of the latter approach, the liver-directed miR-30c analog C2 has demonstrated preclinical activity by coordinately reducing hepatic lipoprotein secretion and lipogenesis while enhancing cholesterol elimination, resulting in reduced LDL-C and atherosclerosis. However, it must be noted that these findings remain preclinical, and further optimization of delivery, pharmacokinetics, safety, and long-term efficacy will be required before clinical evaluation. Continued advances in RNA chemistry, targeted delivery, and genome engineering are expected to further expand the therapeutic landscape for dyslipidemia and cardiovascular disease.

## 1. Introduction

### 1.1. Severe Hypercholesterolemia: A Persistent Unmet Need

**Figure 1:**
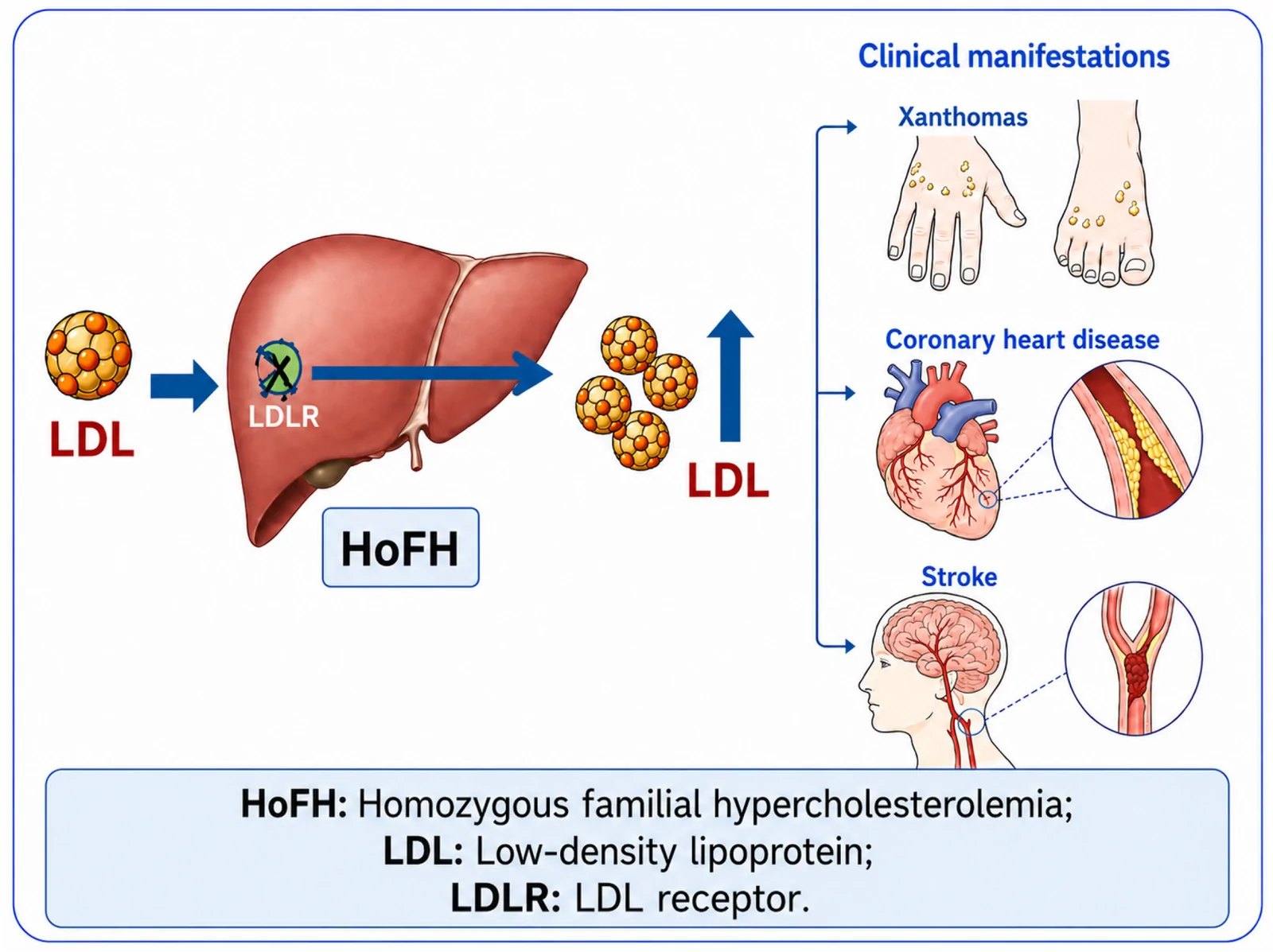
Lack of LDL receptor-induced hypercholesterolemia causes LDL cholesterol to rise. This in turn results in accumulation of fat under the skin in various parts of the body seen as xanthoma. Fat accumulation also occurs in the arteries and blockage of coronary artery leads to heart attack and blockage of carotid and other arteries causes stroke.

Low-density lipoprotein cholesterol (LDL-C) is a causal and modifiable driver of atherosclerotic cardiovascular disease (ASCVD), with a continuous, log-linear relationship between the magnitude of LDL-C lowering and reduction in cardiovascular events [1,2,3]. Familial hypercholesterolemia (FH) is one of the prevalent monogenic metabolic disorders caused by mutations in the low-density lipoprotein receptor (LDLR) gene [4,5,6]. Other gene mutations combined with LDLR mutations cause severe hypercholesterolemia [7]. While hypercholesterolemia has been reported worldwide, it is one of the most common genetic diseases in the Polish population. Recent results [3] showed that as many as 1.6% (1 in 63) of patients with very high cardiovascular disease (CVD) risk have HeFH [8], showing a direct correlation between LDLR and LDL cholesterol leading to CVD. Both homozygous (HoFH) and heterozygous (HeFH) hypercholesterolemia are characterized by a high level of proatherogenic LDL cholesterol that eventually leads to premature coronary heart disease (CHD), xanthomas, and stroke (Figure 1) [9]. Large, randomized trials and Mendelian randomization studies have conclusively demonstrated that earlier, deeper, and sustained LDL-C lowering translates into proportional reductions in myocardial infarction, stroke, and cardiovascular mortality [2,3,9,10]. Accordingly, contemporary U.S. and European guidelines recommend aggressive LDL-C targets—often <55 mg/dL in very high-risk secondary prevention populations and <70 mg/dL in other high-risk patients [10,11].

Both LDL receptor-dependent and -independent therapies have been in clinical use to treat patients with hypercholesterolemia [12], and especially patients with refractory hypercholesterolemia [13]. Thus, despite the availability of statins, ezetimibe, PCSK9 monoclonal antibodies, and newer RNA-based agents, a substantial proportion of patients fail to achieve recommended LDL-C goals in real-world practice [14,15]. This residual burden is most pronounced in patients with HoFH, severe HeFH, and in individuals who are intolerant, partially responsive, or poorly adherent to existing lipid-lowering therapies [16,17,18,19].

Familial hypercholesterolemia represents a particularly high-risk population characterized by lifelong exposure to elevated LDL-C and markedly increased ASCVD risk [18,19]. In HoFH, especially in patients with LDLR-null or near-null mutations, the efficacy of statins and PCSK9-directed therapies is intrinsically limited due to dependence on residual LDLR activity [20,21]. Even with maximal pharmacotherapy and lipoprotein apheresis, many HoFH patients remain far above recommended LDL-C targets and experience progressive ASCVD [20,22]. In HeFH, high baseline LDL-C levels often necessitate long-term combination therapy, increasing cumulative exposure to adverse effects and the likelihood of intolerance or discontinuation [18,20]. Consequently, FH remains an area of substantial residual risk and therapeutic insufficiency, highlighting the need for LDLR-independent approaches that reduce atherogenic lipoprotein production or enhance alternative clearance pathways. The removal of highly atherogenic triglyceride-rich remnant lipoproteins from the circulation is mediated not only by LDL receptor family members but also by heparan sulfate proteoglycans (HSPGs), particularly Syndecan-1, expressed on the surface of hepatocytes. HSPGs bind the positively charged receptor-binding domain of apolipoprotein E (apoE), thereby facilitating hepatic uptake and clearance of apoE-containing remnant lipoproteins, including chylomicron and VLDL remnants, while playing little role in the clearance of mature LDL particles [23,24]. Sulfatase-2 (SULF2) is an extracellular 6-*O*-endosulfatase that regulates this pathway by removing sulfate groups from HSPGs, thereby reducing their affinity for apoE-containing lipoproteins. Inhibition or genetic deletion of SULF2 increases HSPG sulfation, enhances hepatic clearance of triglyceride-rich remnant particles, and lowers plasma triglyceride levels in mice, identifying SULF2 as a negative regulator of remnant lipoprotein clearance [25,26].

### 1.2. Unmet Need in the General Hyperlipidemia Population

Statins remain the cornerstone of LDL-C lowering, yet population-based studies and meta-analyses indicate that 7–9% of treated patients exhibit partial or complete statin intolerance, while 15–20% show inadequate LDL-C reduction despite therapy, and 20–25% discontinue or demonstrate poor adherence over time [21,22,27,28] (Table 1). In the United States, these percentages translate into millions of treated patients with persistently elevated LDL-C, even before accounting for access barriers, polypharmacy, and socioeconomic factors that further limit long-term adherence [29,30].

Real-world experience with PCSK9 monoclonal antibodies has underscored additional limitations (Table 2). Although these agents produce profound LDL-C reductions in clinical trials, their uptake in the U.S. has historically been constrained by prior authorization requirements, high out-of-pocket costs, and prescription abandonment, particularly among Medicare beneficiaries [33,34,35]. Collectively, these factors leave a large population inadequately protected from ASCVD risk despite guideline-directed care.

### 1.3. Rationale for Emerging Nucleic Acid-Based and Network-Level Therapies

These persistent gaps have catalyzed the development of nucleic acid-based therapies, including antisense oligonucleotides, small interfering RNAs (siRNAs), gene therapy, and genome editing strategies aimed at durable or even one-time interventions [39,40,41,42]. The clinical success of siRNA therapies targeting PCSK9 and LPA has validated hepatocyte-directed RNA delivery, long-acting pharmacology, and regulatory pathways for nucleic acid medicines in lipid disorders [40,41,42,43,44].

In parallel, microRNAs (miRNAs) have emerged as master regulators of lipid metabolism, capable of coordinately modulating multiple gene [43,44,45,46,47]. Importantly, human genetics provides compelling proof-of-concept for miRNA-mediated regulation of LDL-C. For example, miR-148a directly represses LDL receptor (LDLR) expression via binding sites in the LDLR 3′ untranslated region (3′UTR) [48]. Individuals in kindred have been identified carrying a ~2.5 kb deletion in the distal LDLR 3′UTR—which removes multiple miRNA-binding sites—exhibit lifelong lower LDL-C and reduced cardiovascular risk, providing a rare human validation of miRNA repression as a powerful way of LDL-lowering [49].

Within this evolving therapeutic landscape, miR-30c has attracted particular attention because it regulates hepatic lipoprotein production through repression of microsomal triglyceride transfer protein (MTTP) while simultaneously suppressing lipid synthesis pathways [47,50,51]. This dual, network-level mechanism offers a biologically distinct and potentially safer approach, especially for patients who are not adequately served by current LDLR-dependent therapies, including HoFH, severe HeFH, and treatment-resistant hypercholesterolemia.

### 1.4. Rationale for New Modalities: Beyond Incremental LDL-C Lowering

The magnitude and persistence of these unmet needs—spanning millions of general hyperlipidemia patients and tens of thousands of high-risk FH patients—have catalyzed the development of new therapeutic modalities. RNA-based drugs (antisense oligonucleotides and siRNAs) have already demonstrated that durable hepatic gene silencing with infrequent dosing is clinically feasible. Gene therapy and genome editing aim to extend this paradigm toward potentially lifelong correction.

Parallel to these advances, microRNAs (miRNAs) have emerged as master regulators of lipid metabolism, offering a complementary strategy that operates at the level of gene networks rather than single targets. However, all efforts relating to therapeutic potential of miRNA technology have suffered setbacks in clinical trials because of the immunogenicity of the drug product in humans; and the silencing of miRNA affecting many pathways [44,52,53].

## 2. Clinically Established RNA-Targeting Therapies for Dyslipidemia

The clinical application of RNA-targeting therapeutics for dyslipidemia represents the culmination of more than three decades of advances in molecular biology, oligonucleotide chemistry, and liver-directed drug delivery. Early skepticism regarding RNA instability, inefficient delivery, and immune activation gradually gave way to clinical feasibility as antisense and RNA interference (RNAi) platforms demonstrated target engagement and lipid lowering in humans [54]. Each successive generation addressed key shortcomings of the prior platform, ultimately leading to clinically viable agents now integrated into cardiovascular prevention.

### 2.1. Mipomersen: First Lipid-Lowering Antisense Therapy

Antisense oligonucleotides (ASOs) were the first RNA-based drugs to enter human trials. These single-stranded oligonucleotides bind complementary mRNA sequences and induce RNase H-mediated degradation or translational repression (30). Chemical innovations such as phosphorothioate backbones and 2′-O-modified sugars markedly improved nuclease resistance and pharmacokinetics, enabling systemic administration by the early 2000s [39]. Mipomersen is a second-generation antisense oligonucleotide (ASO) designed to reduce plasma levels of atherogenic ApoB-containing lipoproteins by selectively inhibiting apolipoprotein B-100 (apoB-100) synthesis in the liver. ApoB-100 is an essential structural protein required for the assembly and secretion of very-low-density lipoprotein (VLDL) particles, which are subsequently metabolized into low-density lipoprotein (LDL). Because each VLDL and LDL particle contains exactly one molecule of apoB-100, suppression of apoB synthesis leads directly to reduced production of circulating atherogenic lipoproteins [55,56,57,58,59,60,61]. Structurally, Mipomersen is a 20-nucleotide phosphorothioate antisense oligonucleotide with 2′-O-methoxyethyl (2′-MOE) modifications at its termini, which enhance nuclease resistance, binding affinity, and pharmacokinetic stability [54,55]. It is complementary to a specific sequence in the coding region of human APOB mRNA. Upon cellular uptake—predominantly by hepatocytes—mipomersen hybridizes with APOB mRNA, forming a DNA–RNA heteroduplex that is recognized and cleaved by RNase H1, an endogenous nuclear enzyme [54]. RNase H-mediated degradation of APOB mRNA prevents translation of apoB-100 protein, thereby limiting the assembly of VLDL particles in the endoplasmic reticulum. Consequently, hepatic secretion of VLDL is reduced, leading to downstream decreases in circulating LDL-C, non-HDL-C, and lipoprotein(a) Lp(a) levels [55,56,57].

### 2.2. Intracellular Lipid Handling, LDLR Independence and Safety Considerations

A key feature of mipomersen’s mechanism is that it is independent of LDL receptor (LDLR) function. Because mipomersen acts upstream by suppressing lipoprotein production rather than enhancing clearance, its lipid-lowering efficacy is preserved in patients with homozygous familial hypercholesterolemia (HoFH), including those with LDLR-null mutations [55]. Clinical studies demonstrated that mipomersen reduces LDL-C by approximately 25–40% in HoFH patients already receiving maximally tolerated lipid-lowering therapy, confirming the LDLR-independent nature of its mechanism [55,56,57]. In addition to LDL-C reduction, mipomersen consistently lowers apoB, non-HDL cholesterol, and Lp(a)—a genetically determined, atherogenic lipoprotein for which few therapies are effective—further supporting its mechanistic breadth [55,56,57].

By inhibiting apoB synthesis and VLDL export, mipomersen alters hepatic lipid trafficking. Reduced VLDL secretion can result in hepatic triglyceride accumulation, as fatty acids that would normally be exported remain within hepatocytes. Indeed, increases in hepatic fat content and elevations in transaminases were observed in both clinical trials and post-marketing surveillance [57,59,62]. These effects are mechanistically consistent with impaired lipoprotein export rather than direct hepatotoxicity. However, unlike regulatory approaches that coordinately suppress lipid synthesis, mipomersen does not downregulate hepatic lipogenesis, leading to an imbalance between lipid input and output. This distinction has become an important conceptual reference point when evaluating newer lipid-lowering strategies, including miRNA-based approaches that aim to suppress lipoprotein secretion while simultaneously modulating lipid synthesis pathways.

## 3. Small Interfering RNA (siRNA) Therapeutics

The conceptual foundation of siRNA therapeutics originates from the discovery of RNA interference (RNAi) in the late 1990s. In 1998, Fire and Mello demonstrated that double-stranded RNA [63] could induce potent, sequence-specific gene silencing in *Caenorhabditis elegans*, a phenomenon they termed RNA interference [63,64]. This landmark finding, recognized by the 2006 Nobel Prize in Physiology or Medicine, revealed a previously unappreciated endogenous gene-regulatory mechanism conserved across eukaryotes.

Subsequent studies rapidly elucidated that RNAi is mediated by short (~21–23 nucleotide) duplex RNAs, now termed small interfering RNAs (siRNAs), which guide the selective degradation of complementary messenger RNAs [64]. The identification of core molecular components—Dicer, Argonaute proteins, and the RNA-induced silencing complex (RISC)—provided the mechanistic framework necessary for therapeutic translation [65,66].

Early enthusiasm for siRNA therapy was tempered by major challenges, including instability in biological fluids, inefficient cellular uptake, off-target effects, and innate immune activation. Over the ensuing two decades, advances in oligonucleotide chemistry and delivery systems transformed RNAi from a biological curiosity into a clinically validated therapeutic platform.

### 3.1. Molecular Mechanism of siRNA-Mediated Gene Silencing

**Figure 2:**
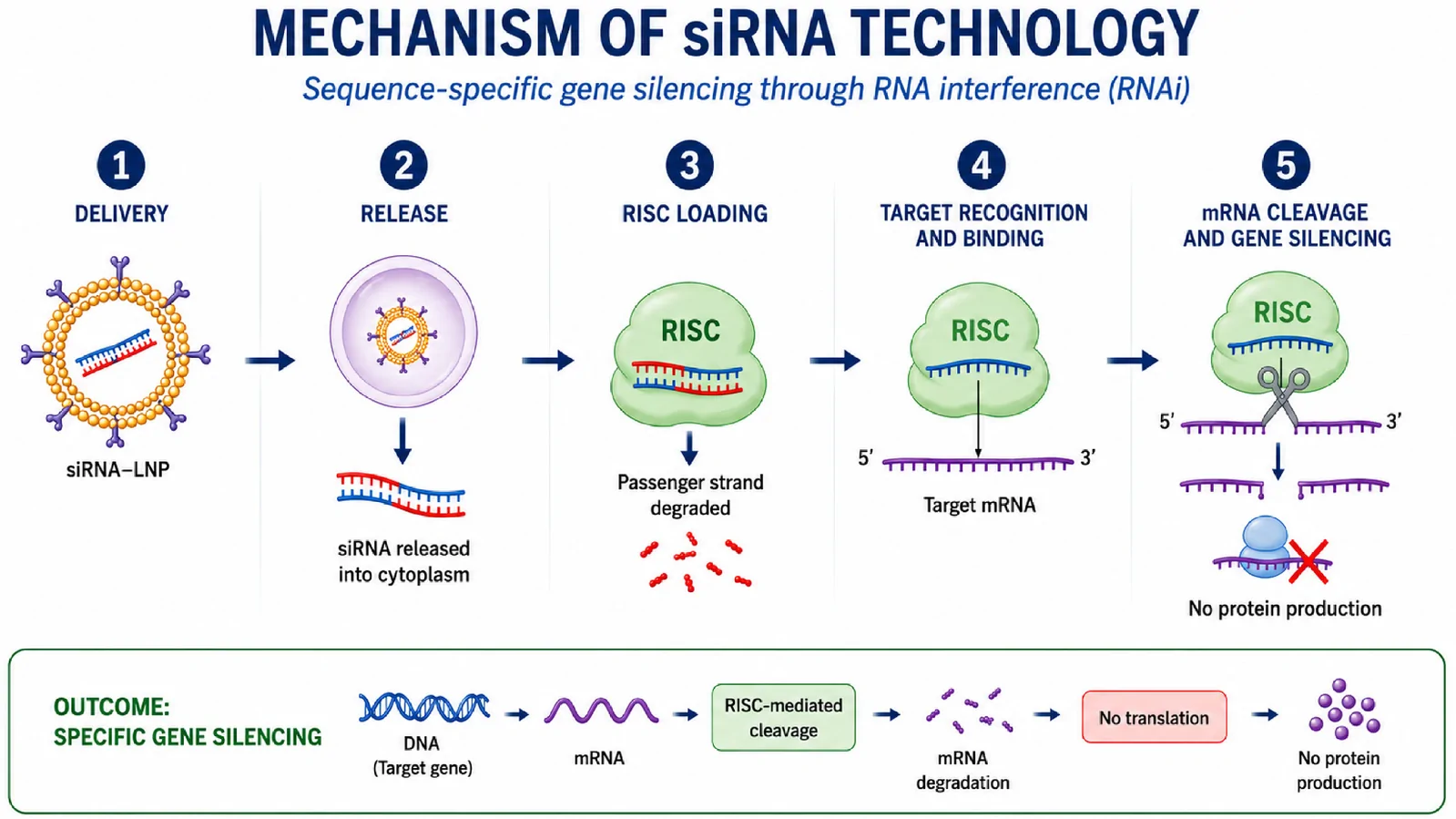
Mechanism of siRNA-mediated gene silencing. Small interfering RNAs (siRNAs) mediate sequence-specific post-transcriptional gene silencing through the endogenous RNA interference (RNAi) pathway. Following delivery into target cells, siRNAs are released into the cytoplasm and incorporated into the RNA-induced silencing complex (RISC). The passenger strand is degraded, while the guide strand directs RISC to complementary target mRNA sequences. Argonaute-mediated cleavage of the target mRNA triggers its degradation, preventing translation and reducing synthesis of the encoded protein. Through catalytic turnover, a single siRNA-loaded RISC can silence multiple mRNA molecules, resulting in potent and highly specific gene knockdown.

Therapeutic siRNAs are synthetically produced, double-stranded RNA molecules designed to mimic endogenous RNAi triggers. Following cellular uptake, siRNAs are incorporated into the RISC, where the passenger strand is discarded and the guide strand is retained by Argonaute-2 (Ago2), the catalytic component of RISC [65]. The guide strand directs RISC to complementary sequences in the target mRNA, resulting in endonucleolytic cleavage of the mRNA at a precise site within the region of complementarity [66] (Figure 2).

Unlike antisense oligonucleotides, which often act in the nucleus via RNase H, siRNA-mediated cleavage occurs predominantly in the cytoplasm and leads to rapid and efficient degradation of the target transcript. Importantly, RISC acts catalytically, enabling a single siRNA molecule to silence multiple mRNA molecules, thereby contributing to the potency and durability of RNAi-based therapies [66].

### 3.2. Evolution of siRNA Delivery Technologies

The primary barrier to siRNA therapeutics has historically been delivery, particularly achieving efficient, tissue-specific uptake while minimizing systemic toxicity. Early systemic administration of naked siRNAs resulted in rapid renal clearance and poor bioavailability [63,64,65,66,67,68,69,70,71,72]. A major breakthrough came with the development of lipid nanoparticle (LNP) delivery systems, which protect siRNAs from degradation and facilitate endosomal escape. LNP technology enabled the first clinical successes in RNAi therapeutics, culminating in regulatory approval of patisiran for hereditary transthyretin amyloidosis [73] (Table 3) (Figure 2). For hepatocyte-targeted therapies—highly relevant to lipid disorders—the introduction of N-acetylgalactosamine (GalNAc) conjugation represented a paradigm shift. GalNAc ligands bind the asialoglycoprotein receptor (ASGPR), which is abundantly expressed on hepatocytes, enabling efficient and selective liver uptake following subcutaneous administration [67]. This strategy dramatically improved safety, convenience, and durability, making chronic siRNA therapy clinically viable (Table 3).

### 3.3. Clinical Validation of siRNA Therapeutics

The success of siRNA technology is underscored by multiple regulatory approvals across diverse indications. In addition to patisiran, other approved siRNA drugs include givosiran (acute hepatic porphyria) [74], lumasiran (primary hyperoxaluria type 1) [75], and inclisiran for hypercholesterolemia [40].

These approvals collectively validated several key principles:

1. **Scalable manufacturing** of chemically modified siRNAs;
2. **Durable gene silencing** with infrequent dosing;
3. **Favorable safety profiles** with appropriate delivery strategies.

### 3.4. siRNA Therapeutics in Lipid Disorders

The most prominent application of siRNA technology in lipid management is inclisiran, a GalNAc-conjugated siRNA targeting proprotein convertase subtilisin/kexin type 9 (PCSK9) mRNA in hepatocytes. By reducing PCSK9 synthesis, inclisiran increases hepatic LDL receptor recycling and enhances LDL clearance from circulation [40].

Clinical trials demonstrated that inclisiran produces ~50% sustained reductions in LDL-C with a dosing regimen of twice yearly following an initial loading phase [40,41,76]. Its long duration of action, consistent efficacy, and favorable tolerability profile led to regulatory approval in multiple regions, establishing siRNA as a mainstream modality for lipid lowering.

Beyond PCSK9, siRNA pipelines have expanded to genetically anchored lipid risk factors. Olpasiran, targeting apolipoprotein(a), has demonstrated profound reductions in lipoprotein(a) [Lp(a)] levels in early-phase trials, addressing a major residual cardiovascular risk factor with limited therapeutic options [77]. Additional siRNA programs targeting angiopoietin-like protein 3 (ANGPTL3) and other lipid regulators are advancing through clinical development, highlighting the versatility of the platform [78,79].

### 3.5. Current Challenges and Future Directions

Despite its success, siRNA therapy is not without limitations. Gene silencing is not permanent, requiring lifelong dosing, and therapeutic effects remain dependent on intact downstream pathways (e.g., functional LDLR for PCSK9 inhibition). Furthermore, siRNA approaches typically target single genes, which may limit efficacy in complex, polygenic metabolic disorders.

Nevertheless, siRNA therapeutics have established a high regulatory and translational benchmark for nucleic acid medicines. Their success has paved the way for complementary modalities—including antisense oligonucleotides, miRNA mimics, gene therapy, and genome editing—each addressing different biological and clinical niches within lipid management.

These advances substantially de-risk the translation of miRNA-based therapeutics, which differ mechanistically by modulating gene networks rather than single transcripts.

## 4. Gene Therapy for Hypercholesterolemia

Gene therapy for hypercholesterolemia has evolved through a stepwise progression from proof-of-concept animal models to early-phase human clinical trials. This trajectory mirrors the broader maturation of the gene therapy field, with successive improvements in vector biology, liver-directed delivery, and immunological risk management. FH, particularly HoFH caused by absent or severely defective LDLR function, has long been considered an archetypal candidate for gene replacement therapy due to its monogenic etiology and hepatic locus of disease pathophysiology [4,5,6,80].

### 4.1. Preclinical Foundations in Animal Models

The conceptual foundation for LDLR gene therapy was established soon after the identification of LDLR mutations as the cause of FH. LDLR-deficient animal models—including LDLR knockout (LDLR^−/−^) mice and Watanabe heritable hyperlipidemic (WHHL) rabbits—recapitulated key features of human FH, including extreme hypercholesterolemia and accelerated atherosclerosis [81,82,83,84,85,86,87,88]. These models provided the first platforms to test whether restoring hepatic LDLR expression could normalize plasma cholesterol levels.

### 4.2. First-Generation Viral Gene Transfer Studies

The earliest proof-of-concept efforts to correct FH at its genetic root emerged in the early to mid-1990s with the use of first-generation adenoviral vectors for hepatic delivery of the LDLR gene. These studies were conducted primarily in LDLR-deficient mice and WHHL rabbits, well-established animal models that closely recapitulate the severe hypercholesterolemia and impaired LDL clearance observed in human FH [40,76,81,82,83,84]. Following systemic administration of recombinant adenoviruses encoding functional LDLR, investigators observed rapid and dramatic reductions in plasma total cholesterol and LDL-C, often exceeding 60–80%, together with markedly enhanced hepatic uptake and clearance of circulating LDL particles [83,84].

Mechanistically, adenoviral-mediated LDLR expression restored receptor-dependent endocytosis of LDL in hepatocytes, thereby directly correcting the fundamental defect underlying FH—namely the absence or dysfunction of hepatic LDLR [83,84]. These experiments provided the first definitive in vivo evidence that hepatic restoration of LDLR expression alone is sufficient to reverse the biochemical phenotype of FH, even in the setting of complete endogenous LDLR deficiency. Importantly, the magnitude and rapidity of cholesterol lowering achieved in these models exceeded that attainable with lipid-lowering pharmacotherapies available at the time, underscoring the transformative potential of gene-based interventions for severe hypercholesterolemia [81,82,83,84].

Despite their striking efficacy [85,86,87,88,89], these first-generation approaches revealed critical translational limitations. Adenoviral vectors triggered robust innate and adaptive immune responses, including activation of Kupffer cells, cytokine release, and development of neutralizing antibodies against viral capsid proteins. These immune responses resulted in transient transgene expression, hepatocellular inflammation, and rapid loss of LDLR expression, with cholesterol levels typically rebounding within days to weeks. In addition, the strong immunogenicity of adenoviral vectors precluded vector re-administration and raised substantial safety concerns, including dose-dependent hepatotoxicity, that ultimately limited clinical applicability [90,91,92,93,94].

Nonetheless, these early studies were foundational for the field of lipid gene therapy. They established several enduring principles that continue to guide contemporary strategies for FH:

(i) Liver is the optimal target organ for LDLR gene replacement;
(ii) Even partial restoration of hepatic LDLR expression can produce profound reductions in plasma LDL-C;
(iii) Durable therapeutic benefit would require vectors capable of long-term transgene expression with minimal immunogenicity [85,90].

These insights directly motivated the subsequent transition to adeno-associated viral (AAV) vectors, which offer improved safety, reduced immunogenicity, and long-term hepatocyte expression, and ultimately ushered in the modern era of liver-directed gene therapy for hypercholesterolemia.

### 4.3. Transition to Second-Generation Gene Therapy

The limitations encountered with first-generation adenoviral vectors made clear that durable correction of FH would require a fundamentally different vector platform. This realization directly catalyzed the transition toward AAV vectors, which emerged in the late 1990s and early 2000s as a second-generation gene delivery system optimized for long-term, liver-directed expression.

Compared with adenoviral vectors, AAV offered several decisive advantages: a markedly improved safety profile, substantially lower innate and adaptive immune activation, and episomal persistence of vector genomes in non-dividing hepatocytes, enabling stable transgene expression over extended periods [88,95]. Importantly, AAV vectors lack viral genes and do not trigger the robust cytotoxic T-cell responses that had curtailed adenoviral approaches, thereby allowing sustained hepatic expression without progressive inflammation or rapid vector clearance. Despite some concerns [96,97,98], new developments provided the biological and translational justification for advancing AAV-based LDLR replacement toward small [94,99] and larger [93] animal models and, ultimately, early-phase clinical evaluation in patients with severe hypercholesterolemia.

### 4.4. Extension to Large-Animal Models: WHHL Rabbits and Translational Relevance

While AAV-mediated LDLR gene transfer demonstrated durable efficacy in rodent models, translation toward human therapy required validation in large-animal systems with lipoprotein metabolism, liver physiology, and immune responses more closely resembling those of humans [100,101,102,103]. In this context, the WHHL rabbit emerged as a particularly valuable model. WHHL rabbits harbor spontaneous LDLR deficiency and develop severe hypercholesterolemia and progressive atherosclerosis, closely paralleling the clinical and biochemical phenotype of homozygous HoFH in humans [83,103,104,105,106,107]. Building on this improved vector biology, multiple independent groups demonstrated that AAV- mediated LDLR gene transfer to the liver of LDLR-deficient animal models produced long-lasting restoration of LDLR expression, resulting in marked and sustained reductions in plasma LDL-C and significant attenuation of atherosclerotic lesion development [88]. In contrast to adenoviral delivery, cholesterol lowering persisted for months to years in these models, closely paralleling the lifespan of hepatocytes [92,94]. AAV-mediated hepatic delivery of functional LDLR in WHHL rabbits resulted in robust and sustained reductions in plasma LDL-C, restoration of LDL clearance, and significant regression or stabilization of established atherosclerotic lesions [88,92,94]. Importantly, LDL-C lowering persisted for extended durations—months to over a year in some studies—reflecting stable episomal persistence of AAV genomes in hepatocytes and confirming the durability of gene expression in a large mammalian liver [93]. These findings provided critical reassurance that the long-term efficacy observed in mice was not species restricted (Table 4).

WHHL rabbit studies enabled systematic evaluation of vector dose–response relationships, identification of thresholds required for meaningful LDL-C reduction, and optimization of liver-specific promoters to maximize hepatocyte expression while minimizing off-target transduction [83,84,85,108].

Complementary studies in nonhuman primates further strengthened translational confidence. Although primates do not fully recapitulate FH genetics, AAV liver tropism, promoter activity, and long-term transgene expression kinetics could be evaluated in a system closely aligned with human hepatic architecture and immune regulation [109,110,111,112]. These studies validated vector manufacturing scalability, dosing strategies, and clinical monitoring paradigms that would later be adopted in first-in-human gene therapy trials for metabolic and coagulation disorders.

Collectively, large-animal AAV-LDLR studies bridged the critical gap between rodent proof-of-concept and human clinical translation. They demonstrated that LDLR gene replacement could durably lower LDL-C and modify atherosclerotic disease in a physiologically relevant setting, while simultaneously informing vector design, dosing, liver specificity, and safety monitoring strategies essential for regulatory advancement. These data formed the experimental foundation upon which early-phase AAV-based gene therapy trials for severe hypercholesterolemia were rationally pursued.

### 4.5. Lessons Learned from Large-Animal Studies: Nonclinical Pharmacology and Toxicology

Large-animal studies provided critical insights that extended beyond efficacy and were essential for advancing AAV-based LDLR gene therapy toward clinical testing. Collectively, these investigations established a translational framework encompassing dose selection, tissue specificity, durability, and safety, all of which are central to regulatory evaluation.

First, studies in mice, WHHL rabbits and nonhuman primates confirmed that therapeutically meaningful LDL-C lowering could be achieved with partial hepatic LDLR expression, reducing the need for supraphysiologic transgene levels and thereby widening the therapeutic window. This finding informed conservative dose-escalation strategies for clinical trials while preserving efficacy [84,87,88].

Second, large-animal models clarified the biodistribution profile of hepatotropic AAV serotypes, demonstrating predominant liver transduction with limited extrahepatic exposure when liver-specific promoters were employed. These data supported the rationale for liver-directed expression as both an efficacy- and safety-enhancing design principle [83,84,88,113].

Third, immune responses emerged as a manageable—but nontrivial—consideration. While capsid-directed humoral immunity and transient transaminase elevations were observed, they were substantially attenuated compared with first-generation adenoviral vectors and could be monitored and mitigated through dose control, patient selection, and immunomodulatory strategies [95,96,97,98]. Importantly, no progressive liver injury or loss of transgene expression was observed in well-controlled settings.

Finally, prior to first-in-human testing, formal good laboratory practice (GLP) pharmacology and toxicology studies were conducted in LDLR-deficient mouse models and nonhuman primates using clinical-grade vectors (e.g., AAV8.TBG.hLDLR). These studies systematically evaluated vector biodistribution, dose-dependent hepatotoxicity, innate and adaptive immune activation, germline transmission risk, and vector shedding, thereby establishing a safety margin suitable for regulatory submission and clinical translation [93].

Together, these large-animal and GLP toxicology studies transformed AAV-LDLR gene therapy from a proof-of-concept into a regulatorily credible therapeutic strategy, providing the quantitative and qualitative data necessary to justify first-in-human trials for severe hypercholesterolemia.

### 4.6. LDLR Gene Replacement in HoFH

HoFH represents the strongest use case for LDLR gene replacement, as patients with LDLR-null or near-null mutations derive limited benefit from LDLR-dependent therapies such as statins or PCSK9 inhibitors [12,16,18,21,22]. Gene therapy offers a mechanistically restorative approach by reintroducing functional LDLR directly into hepatocytes.

#### 4.6.1. Transition to First-in-Human AAV-LDLR Clinical Evaluation

The convergence of durable efficacy in rodent and large-animal models, together with comprehensive GLP pharmacology and toxicology datasets, established a sufficiently robust translational foundation to support first-in-human testing of liver-directed LDLR gene therapy. This progression followed a classical regulatory logic: demonstration of disease modification in relevant animal models, characterization of dose–response relationships, confirmation of liver-restricted biodistribution, and establishment of an acceptable safety margin under GLP conditions.

This rationale culminated in the initiation of a Phase I/IIa open-label, dose-escalation clinical trial evaluating AAV8.TBG.hLDLR in adults with HoFH (ClinicalTrials.gov identifier NCT02651675) [114]. The study employed a single intravenous administration, reflecting the intended one-time therapeutic paradigm of in vivo gene therapy. Primary endpoints were focused on safety and tolerability, including hepatic adverse events, immune responses to the AAV capsid or transgene product, and laboratory abnormalities. Secondary endpoints included changes in plasma LDL-C, apoB levels, and assessment of durability of LDLR expression and lipid lowering over extended follow-up [82,84].

Although peer-reviewed efficacy data remain limited, the initiation and continuation of this study represent a landmark translational milestone. Importantly, the trial validated the regulatory feasibility of liver-directed LDLR gene therapy in FH, including vector manufacturing under current good manufacturing practice (cGMP) conditions, clinical-grade dosing, and implementation of long-term patient monitoring frameworks required for in vivo gene transfer studies. Long-term follow-up is ongoing, consistent with regulatory guidance mandating extended surveillance—often 5–15 years—for delayed adverse events, durability of expression, and oncogenic risk associated with viral gene therapy products [93,115,116,117].

**Table 4:** Preclinical-to-clinical evolution of gene therapy for hypercholesterolemia. Chronological progression of LDLR gene therapy for hypercholesterolemia.

Period	Model/Milestone	Key Findings	Translational Significance
1980s–1990s	LDLR^−/−^ mice; WHHL rabbits	Severe hypercholesterolemia and atherosclerosis	Established disease models [83,101,118]
Early 1990s	Adenoviral LDLR transfer (rodents)	Rapid LDL-C reduction; transient expression	Proof of principle; immunogenicity limits [85,119]
2000–2005	AAV-LDLR in LDLR^−/−^ mice	Durable LDLR expression; ↓ LDL-C; ↓ plaques	Established AAV feasibility [120,121]
2006–2012	AAV-LDLR in Mice and WHHL rabbits	Sustained LDL-C lowering in large animals	Strengthened human relevance [99,122]
2013–2017	GLP tox studies (AAV8.TBG.hLDLR)	Acceptable safety margins	Enabled IND filing [93,117]
2016–2020	Phase I/IIa HoFH trial (NCT02651675)	Safety-focused human translation	First clinical test of LDLR gene replacement [114]

#### 4.6.2. Regulatory Risk–Benefit Considerations for AAV-LDLR Gene Therapy

From a regulatory perspective, AAV-mediated LDLR gene therapy occupies a distinct risk–benefit niche shaped by disease severity, unmet medical need, and availability of alternative therapies. HoFH is a life-threatening condition characterized by lifelong exposure to extreme LDL-C levels and markedly elevated risk of premature atherosclerotic cardiovascular disease. Despite advances in lipid-lowering pharmacotherapy, many patients remain inadequately controlled, particularly those with LDLR-null mutations. This clinical context supports consideration of higher-risk, potentially transformative interventions, including gene therapy.

Balanced against these risks is the potential for durable, LDLR-independent correction of the underlying metabolic defect, achieved with a single administration and without the burden of lifelong drug adherence. Importantly, nonclinical and early clinical experience suggests that partial hepatic LDLR expression may be sufficient to achieve clinically meaningful LDL-C reductions, potentially mitigating dose-related toxicity while preserving benefit. As such, regulatory assessment emphasizes a risk-adaptive framework, wherein the severity of HoFH and lack of alternatives justify controlled exposure to gene therapy-associated risks, provided that rigorous safety monitoring and long-term follow-up are maintained.

Collectively, first-in-human AAV-LDLR trials mark a critical inflection point in the evolution of lipid therapeutics—bridging decades of mechanistic insight and nonclinical validation into a new therapeutic class with the potential to redefine treatment paradigms for the most severe forms of hypercholesterolemia.

#### 4.6.3. Platform Constraints and Translational Challenges

Despite strong biological rationale and preclinical success, several constraints continue to shape FH gene therapy development:

1. **AAV packaging capacity (~4.7 kb)** limits regulatory flexibility and necessitates optimized promoters and codon engineering [96].
2. **Pre-existing neutralizing antibodies** to AAV capsids exclude a subset of patients and complicate re-dosing strategies [97].
3. **Dose-dependent immunotoxicity**, including hepatotoxicity and complement activation, has emerged as a central risk in systemic AAV programs [85,98].
4. **Durability uncertainty**, particularly in the regenerating liver, requires long-term monitoring for waning expression and rare genotoxic events [113,115].
5. **Patient selection**, based on LDLR genotype and residual function, is critical to maximizing benefit-risk balance [19,20,97].

## 5. Genome Editing for Hypercholesterolemia

Genome editing aims to achieve durable suppression or correction of lipid-regulating genes after a single administration, offering the possibility of long-lived lowering of atherogenic lipoproteins. This therapeutic concept is especially compelling for severe hypercholesterolemia, where lifelong exposure to elevated LDL-C drives premature ASCVD. Among potential target organs, the liver has emerged as the leading site for cardiometabolic genome editing because hepatocytes can be efficiently targeted using LNPs and other hepatotropic delivery systems that enable systemic intravenous dosing with high hepatic enrichment and limited off-target exposure.

At the same time, translation of genome editing into preventive cardiometabolic disease is held to an exceptionally high safety and regulatory bar. Unlike oncology or rapidly fatal genetic disorders, hypercholesterolemia is common, often manageable with chronic therapies, and treated largely for prevention. Accordingly, durable benefit must be weighed against (i) the permanence of genomic modification, (ii) limited reversibility or re-dosing options, and (iii) the requirement for long-term surveillance to detect delayed or rare adverse events that may emerge years after treatment [123].

### 5.1. CRISPR–Cas9 and the Evolution of Genome and Base Editing

The development of Clustered regularly interspaced short palindromic repeat (CRISPR) and its associated protein (Cas-9) (CRISPR–Cas9) technology represents one of the most transformative advances in modern molecular biology, enabling precise, efficient, and programmable genome editing (Table 5). In 2013, two groups independently demonstrated that CRISPR–Cas9 could be adapted for targeted genome editing in mammalian cells [124,125]. This system relies on Cas9-induced double-strand DNA breaks (DSBs), which are repaired by cellular pathways such as non-homologous end joining (NHEJ) or homology-directed repair (HDR), enabling gene disruption or precise sequence insertion. Further development of CRISPR–Cas systems [126,127] enabled programmable genomic targeting dramatically simplifying gene modification compared with earlier nuclease platforms [128,129]. Further refinement led to prime editing, also developed by Liu’s group [127,130], which enables targeted insertions, deletions, and all 12 possible base substitutions without requiring donor DNA templates or DSBs. Prime editors combine Cas9 nickase with reverse transcriptase and a prime editing guide RNA (pegRNA), significantly expanding the scope of genome editing. Subsequent innovations—most notably base editors and related precision editors [131,132], further advanced the field by enabling targeted nucleotide conversion without creating double-strand breaks, thereby reducing reliance on error-prone DNA repair pathways and improving suitability for in vivo applications [133,134].

LNPs have emerged as a leading non-viral delivery platform for genome editing, particularly for targeting the liver, owing to their ability to efficiently encapsulate and deliver mRNA and guide RNAs with favorable safety profiles [145,146]. Further advances in LNP delivery technology transformed genome editing from an ex vivo or local intervention into a systemically deliverable modality [147,148]. LNPs capable of encapsulating mRNA, guide RNAs, and base-editing components demonstrated efficient hepatocyte uptake following intravenous administration, enabling reproducible in vivo editing in animal models [149,150] and, ultimately, opening a door for therapy in humans. Together, these advances [151,152,153] established a credible technical foundation for liver-directed genome editing in lipid disorders.

### 5.2. Editing PCSK9: The Flagship Pathway for LDL-C Lowering

Genome editing has emerged as a promising therapeutic strategy for cardiovascular disease, particularly for the treatment of hypercholesterolemia through permanent modification of lipid-regulating genes [154].

LDL receptor has already been recognized as one of the first targets to manage hypercholesterolemia using genome editing technology. Zhou et al. [155] designed the AAV-CRISPR/Cas9 to correct the point mutation in the LDLR gene in hepatocytes and delivered subcutaneously into *Ldlr^E208X^* mice. This led to significant reductions in LDL cholesterol and triglycerides in the serum that resulted in smaller atherosclerotic plaques in the aorta and a lower degree of macrophage infiltration. Another target that emerged as the first flagship target for cardiometabolic genome editing turned out to be PCSK9 based on robust data obtained from human genetics, monoclonal antibodies, and siRNA therapies, all demonstrating that restoration of LDLR expression and sustained PCSK9 suppression produce robust LDL-C lowering with favorable clinical outcomes. Early efforts using CRISPR–Cas9 demonstrated that targeting hepatic *PCSK9* could significantly reduce circulating LDL-C levels by enhancing LDL receptor availability. In mouse models, CRISPR-mediated disruption of *PCSK9* resulted in sustained reductions in plasma cholesterol [156,157], a finding later translated to non-human primates with long-lasting lipid lowering [158]. More recently, base editing approaches have provided a safer and more precise alternative by avoiding double-strand DNA breaks. A landmark preclinical study showed that LNP-delivered base editing of PCSK9 in nonhuman primates achieved high-efficiency editing in hepatocytes, resulting in durable reductions in circulating PCSK9 protein and LDL-C after a single administration. Notably, adenine base editors have been used to introduce loss-of-function mutations in *PCSK9* in vivo, achieving durable LDL-C reductions in primates with minimal off-target effects [135]. Subsequent nonclinical studies reinforced this translational case by demonstrating reproducible PCSK9 editing, sustained lipid lowering, and manageable safety profiles across different editor designs and dosing paradigms in primates [136].

Collectively, genome and base editing technologies hold transformative potential for one-time, lifelong treatment of cardiovascular disease by addressing its genetic root causes, although long-term safety, delivery efficiency, and off-target risks remain key considerations.

### 5.3. First-in-Human Cardiometabolic Editing Programs

The translatability of in vivo genome editing in non-human primates to suppress PCSK9 activity and sustained lowering of LDL cholesterol has been established [135,136]. These advances in non-human primates helped gather in vivo genome editing technology knowledge and associated risks to design early-phase CRISPR-based therapies targeting *PCSK9* for patients with familial hypercholesterolemia. Clinical translation has begun with first-in-human PCSK9 base-editing programs, including VERVE-101 (NCT05398029) [140] and next-generation candidates such as VERVE-102 (NCT06164730) [143]. These programs reflect iterative optimization of editor chemistry, delivery efficiency, and tolerability, informed by accumulating nonclinical and early clinical experience [159]. At this stage, clinical development emphasizes careful dose escalation, intensive monitoring of hepatic safety, immune responses, and off-target effects, and extended follow-up to characterize durability and late-emerging events. Importantly, these trials represent a conceptual shift: genome editing is being evaluated not merely as a rescue therapy, but as a potential preventive intervention in high-risk populations—a distinction that fundamentally shapes regulatory scrutiny and benefit–risk assessment [159].

Wan et al. [160] carried out in vivo gene therapy in heterozygous hypercholesterolemic subjects (*n* = 6) with YOLT-101, an investigational genome editing product that uses adenine base-editing technology. It was delivered via GalNAc-modified lipid nanoparticles to target liver and inactivate PCSK9. Interim results from an ongoing clinical trial evaluating primary (safety and tolerability) and secondary (lowering of PCSK9 and LDL-C levels) outcomes of a single intravenous dose of YOLT-101 in adults with heterozygous familial hypercholesterolemia and uncontrolled LDL-C showed dose-dependent and durable reductions of 74.4% and 52.3% at 24 weeks in circulating PCSK9 and LDL-C, respectively [160,161].

### 5.4. Editing ANGPTL3 and Additional Targets: LDLR-Independent Lipid Lowering

ANGPTL3 is a liver-derived regulator of lipoprotein metabolism, and unlike LDLR-dependent approaches, its inhibition can lower atherogenic lipids through a mechanism that remains relevant even when LDL receptor activity is impaired. That is particularly important for difficult populations such as homozygous familial hypercholesterolemia and mixed dyslipidemias. The target is also unusually well de-risked biologically: natural human deficiency appears cardioprotective rather than harmful [162,163], and approved or late-stage non-editing ANGPTL3 drugs have already shown that substantial pharmacologic suppression is tolerable and effective [164,165]. Thus, ANGPTL3 has gained attention as a complementary genome-editing target because its inhibition lowers atherogenic lipoproteins through pathways that are partly independent of LDLR function. This feature is particularly relevant for HoFH and severe heterozygous FH, where LDLR-based therapies are intrinsically limited.

Two main editing strategies have advanced for ANGPTL3: nuclease-based CRISPR-Cas9 knockout and adenine base editing. On the base-editing side, Verve’s VERVE-201 program [150] uses an adenine base editor and guide RNA delivered by a GalNAc-lipid nanoparticle designed to route uptake through the hepatocyte asialoglycoprotein receptor rather than relying on LDLR-mediated uptake. In nonhuman primates, Verve reports [150] mean whole-liver ANGPTL3 editing of about 63% at the higher tested dose, with mean blood ANGPTL3 reduction of 96% and durability out to 22 months; in LDLR-deficient primates, the program reported about 60% liver editing with mean LDL-C reduction of 46% and triglyceride reduction of 54%. A separate 2025 peer-reviewed study using LNP-delivered adenine base editing in mice achieved over 60% liver editing with durable reductions in ANGPTL3, LDL-C, and triglycerides for at least 100 days, extending to 191 days under dietary challenge [142]. These results collectively show that durable liver editing of ANGPTL3 is technically achievable and biologically potent.

### 5.5. Lessons from First-in-Human Systemic In Vivo Editing (Cross-Indication)

VERVE-201 is the first ANGPTL3 base-editing medicine known to have entered human testing. Verve states that it is conducting the **Phase 1b** trial in adults with refractory hypercholesterolemia, and company materials say the first participant was dosed in November 2024. The study is a single-ascending-dose, open-label trial, with endpoints including safety, pharmacokinetics, blood ANGPTL3 protein, and LDL-C change; third-party registries and Lilly materials identify the study as NCT06451770, with an estimated enrollment of 36 participants. As of early 2026, I could find evidence that the trial remains active, but I did not find peer-reviewed human efficacy data for VERVE-201 yet, so the program appears to be clinically underway but still pre-readout in the published literature.

The most advanced published human evidence for ANGPTL3 editing comes from CTX310, CRISPR Therapeutics’ LNP-delivered CRISPR-Cas9 nuclease program. The ongoing phase 1 trial is registered as NCT07491172/ACTRN12623000809639 [166] and is designed as a first-in-human, open-label, ascending single-dose study in refractory dyslipidemias. According to the trial registry, the study evaluates single intravenous doses from 0.1 to 0.8 mg/kg, with follow-up to 12 months and a target sample size of 24. The first published phase 1 report, presented at AHA 2025 and summarized by the ACC from the NEJM publication [167], found no dose-limiting toxicities and no serious adverse events judged related to CTX310. At higher doses, the lipid effects were clinically meaningful: at 0.8 mg/kg, mean ANGPTL3 fell 73.2%, LDL-C 48.9%, triglycerides 55.2%, ApoB 33.4%, and non-HDL cholesterol 49.8%; at 0.6 mg/kg, triglycerides fell 62.0% and LDL-C 39.2%. One sudden death 179 days after treatment in a participant treated at 0.1 mg/kg was adjudicated as unrelated. These data mark the first clear proof-of-concept that in vivo ANGPTL3 genome editing can lower atherogenic lipids in humans after a single infusion.

Longer-term and expanded experience in cardiometabolic disorders will further reinforce the feasibility of durable target suppression after one administration, while also emphasizing the importance of rigorous hepatic safety monitoring, immune surveillance, and long-term follow-up in systemic editing programs [167]. These lessons will directly inform cardiometabolic editing strategies, where safety expectations are even higher. The Chronological milestones in genome editing relevant to hypercholesterolemia are summarized in Table 5.

### 5.6. Safety and Regulatory Considerations for Cardiometabolic Genome Editing

Because hypercholesterolemia is common and often managed effectively with chronic medications, regulatory and ethical frameworks demand unusually rigorous risk minimization for one-time genome editing interventions [150,167].

**Off-target editing and specificity.** Developers must quantify off-target modifications using complementary approaches, combining in silico prediction with empirical genome-wide assays at clinically relevant exposures [123,168]. While base editors reduce risks associated with double-strand breaks, they can introduce unintended base conversions in DNA—and potentially RNA—necessitating comprehensive profiling [123,159,168].

**On-target unintended consequences.** Even at the intended locus, editing may generate bystander edits, unexpected insertions/deletions, or larger genomic rearrangements, depending on editor architecture and sequence context. These risks drive the need for deep locus-specific sequencing and broader genomic integrity assessments in nonclinical packages [123,159,168].

**Immunogenicity.** Cas proteins, editor components, and LNP formulations can elicit immune responses. Clinically, transient inflammatory reactions and liver enzyme elevations are recognized monitoring priorities for hepatocyte-targeted nucleic acid delivery and editing programs [123,159,168].

**Biodistribution and unintended exposure.** Although liver tropism is high, some extrahepatic distribution may occur. Regulators expect quantitative biodistribution data to inform tissue-specific risk assessment, clinical monitoring, and reproductive safety strategies.

**Germline considerations.** Programs must demonstrate that germline transmission risk is minimized or absent and incorporate appropriate reproductive toxicology strategies, depending on biodistribution and patient population.

**Durability versus controllability.** While durability is the therapeutic goal, irreversibility raises concern for rare delayed adverse events. Consequently, long-term follow-up is a core regulatory expectation for genome editing products [135,142,169].

### 5.7. Core Regulatory Expectations

Regulators require fit-for-purpose nonclinical biodistribution studies in line with ICH S12 to guide starting dose selection, clinical monitoring, and long-term risk assessment. IND-enabling packages must also include robust CMC controls, addressing identity, purity, potency, and formulation attributes of both editor components and LNPs, consistent with FDA gene therapy guidance. Because genome editing platforms evolve rapidly, comparability and lifecycle management considerations are particularly important.

Extended long-term follow-up (LTFU) is expected for products designed to achieve long-lasting or permanent effects, with monitoring often extending years beyond active trial participation. In Europe, EMA gene therapy frameworks similarly emphasize long-term safety, risk management plans, and post-authorization surveillance.

### 5.8. Translational Implications Specific to Hypercholesterolemia

For hypercholesterolemia, acceptable risk is strongly influenced by disease severity, residual risk despite optimal therapy, and the preventive nature of treatment. Accordingly, cardiometabolic genome editing programs are expected to (i) prioritize high-risk populations with substantial unmet need (e.g., severe FH), (ii) generate unusually robust nonclinical specificity and biodistribution datasets, and (iii) implement long-term monitoring aligned with gene therapy regulatory guidance.

## 6. miRNAs as Network Regulators of Cholesterol Homeostasis: Biology, Mechanisms, and Human Proof-of-Concept

miRNAs are small (~22-nucleotide) noncoding RNAs that regulate gene expression post-transcriptionally and have emerged as fundamental regulators of metabolic homeostasis. Unlike classical transcription factors or single-enzyme drug targets, miRNAs act as network regulators, simultaneously modulating multiple transcripts within interconnected pathways. This systems-level control is particularly relevant to lipid and cholesterol metabolism, where synthesis, lipoprotein assembly, clearance, and efflux must remain tightly coordinated over a lifetime.

### 6.1. Discovery and Conceptual Evolution of miRNAs

miRNAs were first discovered in the early 1990s through genetic studies in Caenorhabditis elegans. In 1993, Ambros and colleagues identified lin-4, a small RNA that regulated developmental timing by binding to the 3′ untranslated region (3′UTR) of its target mRNA, lin-14, introducing a novel RNA-based mode of gene regulation [170].

The field expanded decisively with the discovery of let-7 in 2000, which was shown to be conserved across species, establishing miRNAs as an evolutionarily ancient and widespread regulatory mechanism [171]. Subsequent large-scale cloning and sequencing efforts identified hundreds of miRNAs in animals and humans, revealing that miRNAs are pervasive regulators rather than biological curiosities [171,172,173].

By the mid-2000s, it became clear that miRNAs regulate diverse biological processes—including development, differentiation, metabolism, and stress responses—and that dysregulation of miRNAs contributes to human diseases such as cancer, cardiovascular disease, and metabolic disorders [43,44].

### 6.2. miRNA Biogenesis and Molecular Mechanism of Action

**Figure 3:**
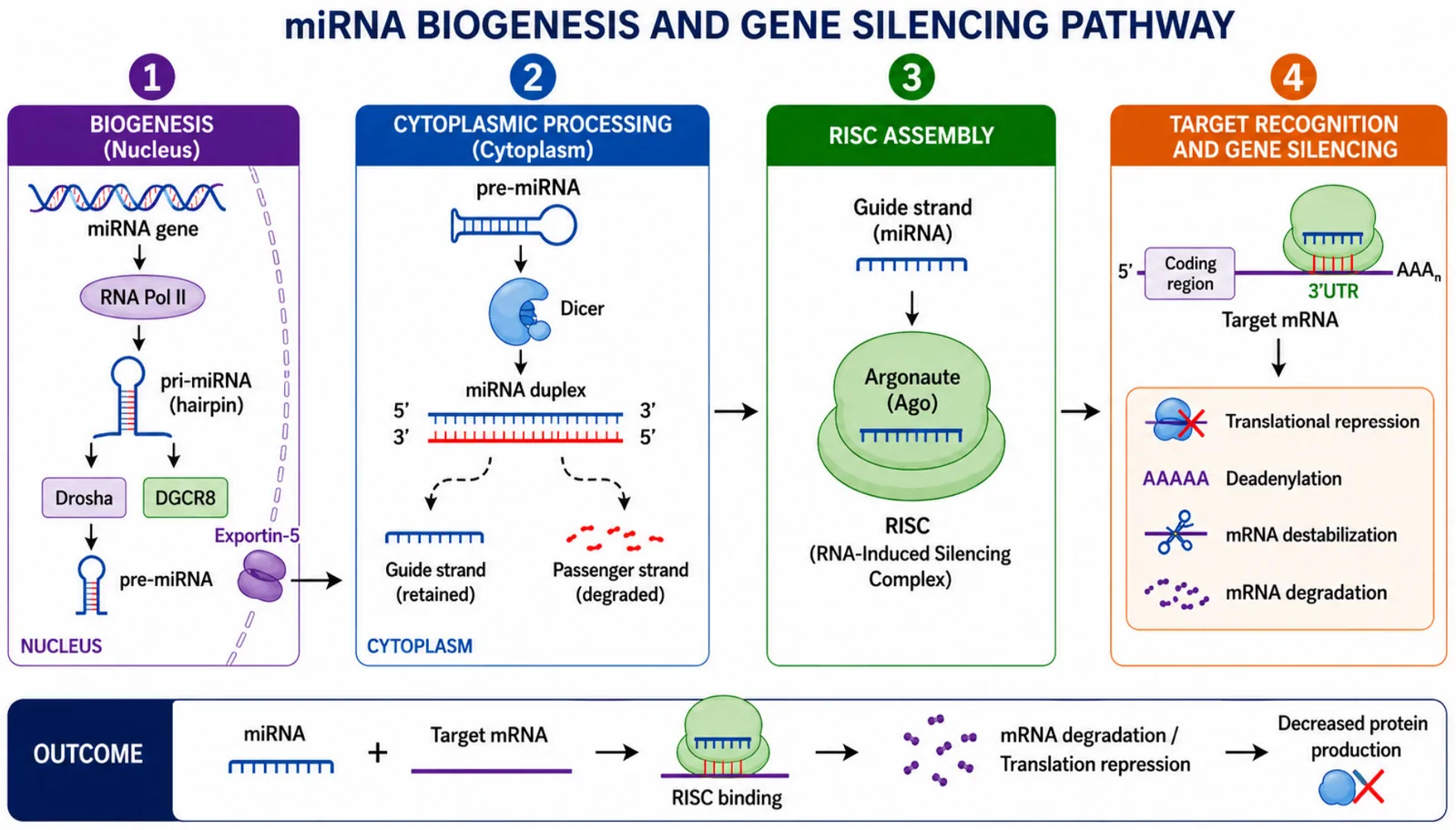
***Panel 1***. miRNA Biogenesis in the Nucleus. miRNA genes are transcribed by RNA polymerase II to generate primary miRNA transcripts (pri-miRNAs). The Drosha–DGCR8 microprocessor complex cleaves pri-miRNAs into precursor miRNAs (pre-miRNAs), which are subsequently exported to the cytoplasm by Exportin-5. ***Panel 2***. Cytoplasmic Processing of pre-miRNAs. In the cytoplasm, Dicer cleaves pre-miRNAs to generate a mature miRNA duplex. The passenger strand is degraded, while the guide strand is retained for incorporation into the RNA-induced silencing complex (RISC). ***Panel 3***. RISC Assembly. The guide miRNA strand is loaded onto an Argonaute-containing RISC, forming the active silencing machinery responsible for recognizing and regulating target mRNAs. ***Panel 4***. Target Recognition and Gene Silencing. The miRNA-RISC complex binds complementary sequences, typically within the 3′ untranslated region (3′UTR) of target mRNAs. This interaction promotes translational repression, mRNA deadenylation, destabilization, and degradation, resulting in reduced protein expression.

Canonical miRNA biogenesis begins with transcription of primary miRNA transcripts (pri-miRNAs), usually by RNA polymerase II. In the nucleus, pri-miRNAs are processed by the Drosha–DGCR8 microprocessor complex into ~70-nucleotide precursor miRNAs (pre-miRNAs) [46]. These are exported to the cytoplasm, where Dicer cleaves them into ~22-nt miRNA duplexes [174]. One strand of the duplex is incorporated into the Argonaute-containing RNA-induced silencing complex (RISC), while the passenger strand is degraded (Figure 3). The mature miRNA guides RISC to partially complementary sequences—most often within the 3′UTR of target mRNAs—resulting in translational repression and/or mRNA destabilization [68,175].

Because miRNA–mRNA recognition relies on partial complementarity, a single miRNA can regulate dozens to hundreds of transcripts, enabling coordinated control of entire pathways rather than binary on/off regulation [44,52] (Figure 3).

### 6.3. miRNAs as Network Regulators of Lipid and Cholesterol Metabolism

Cholesterol homeostasis is governed by a tightly integrated network involving hepatic cholesterol synthesis, lipoprotein assembly and secretion, LDL receptor-mediated uptake, and HDL-dependent reverse cholesterol transport. miRNAs intersect with each of these nodes and provide post-transcriptional fine-tuning of lipid flux [44,45,176].

Unlike coding mutations, which often produce abrupt functional loss, miRNAs act as rheostats, subtly modulating gene expression. This property is especially relevant to hypercholesterolemia and ASCVD, where modest but sustained changes in LDL-C over decades translate into large differences in cardiovascular risk [45,177].

### 6.4. Canonical microRNAs Governing Cholesterol Homeostasis

A defining feature of miRNA biology in lipid metabolism is that individual miRNAs rarely act on a single gene or pathway. Instead, they function as network regulators, coordinating multiple nodes within cholesterol synthesis, transport, efflux, and lipoprotein metabolism. This property is particularly relevant for complex, polygenic disorders such as hypercholesterolemia and atherosclerotic cardiovascular disease, where modest, coordinated shifts across pathways may yield greater and more durable benefit than strong perturbation of a single target. The miRNAs discussed below—miR-33, miR-144, and miR-122—provide instructive precedents that frame the therapeutic rationale for miR-30c-based approaches.

#### 6.4.1. miR-33 Family: Linking Sterol Sensing to Cholesterol Efflux and HDL Biogenesis

The miR-33 family (miR-33a and miR-33b) represents one of the clearest examples of how miRNAs integrate transcriptional and post-transcriptional regulation in cholesterol metabolism. miR-33a and miR-33b are encoded within introns of SREBF2 and SREBF1, respectively, genes that encode sterol regulatory element-binding proteins (SREBPs), the master transcriptional regulators of cholesterol and fatty-acid biosynthesis. This genomic organization creates a tightly coupled regulatory circuit in which activation of cholesterol synthesis is coordinated with suppression of cholesterol efflux [178,179,180].

Functionally, miR-33 directly represses the ATP-binding cassette transporters ABCA1 and ABCG1, key mediators of cholesterol efflux to apolipoprotein A-I and HDL particles [179,180]. By limiting ABCA1/ABCG1 expression, miR-33 constrains HDL biogenesis and reverse cholesterol transport, favoring intracellular cholesterol retention during sterol scarcity. While physiologically adaptive, this mechanism becomes maladaptive in dyslipidemic states, where persistent miR-33 activity contributes to impaired cholesterol efflux and foam-cell formation leading to reductions in atherosclerotic lesion formation in LDLR-deficient mice [181].

Pharmacologic inhibition of miR-33 has yielded consistent lipid effects across species. In rodent models, antisense inhibition of miR-33 increases ABCA1 expression in liver and macrophages, enhances cholesterol efflux, and raises circulating HDL-C levels [179,180,181]. Importantly, translation to nonhuman primates demonstrated increased HDL-C and reduced VLDL triglycerides following miR-33a/b inhibition, providing a crucial higher-species validation of therapeutic plausibility [181].

#### 6.4.2. miR-144: Post-Transcriptional Control of ABCA1 Within Bile Acid–Cholesterol Signaling Axes

miR-144 adds an additional layer of regulation to cholesterol efflux pathways by directly repressing ABCA1 in hepatocytes and macrophages [182]. While miR-33 is embedded within sterol-sensing transcriptional programs, miR-144 is notable for its regulation by nuclear receptor signaling, particularly the farnesoid X receptor (FXR), which is activated by bile acids.

FXR activation induces miR-144 expression, thereby linking bile acid flux and hepatic metabolic state to post-transcriptional repression of cholesterol efflux [183,184]. Experimental overexpression of miR-144 reduces ABCA1 levels, impairs macrophage cholesterol efflux, and lowers plasma HDL-C, whereas miR-144 inhibition produces reciprocal effects. Through this mechanism, miR-144 integrates hepatobiliary signaling with systemic cholesterol handling.

Although miR-144 has not yet advanced to clinical development, its biology reinforces the principle that miRNA-based interventions influence interconnected metabolic pathways, a theme central to the rationale for miR-30c-based therapy [47,50,51].

#### 6.4.3. miR-122: Hepatic Lipid Metabolism and Clinical Validation of miRNA Targeting

miR-122 occupies a unique position in miRNA biology as the dominant hepatic miRNA, comprising approximately 70% of total miRNA content in the liver. Unlike miR-33 and miR-144, which exert relatively focused effects on cholesterol efflux, miR-122 regulates broad hepatic metabolic programs, influencing cholesterol biosynthesis, fatty-acid metabolism, and lipoprotein production through coordinated effects on multiple target genes [182,185].

In animal models, antisense inhibition of miR-122 results in substantial reductions in plasma cholesterol and triglycerides, accompanied by downregulation of lipogenic and cholesterologenic gene expression [186]. These effects provided early evidence that targeting a single, highly abundant miRNA could produce systemic metabolic benefits.

Crucially, miR-122 also established the first clinical proof-of-concept for miRNA therapeutics. Miravirsen, an antisense inhibitor of miR-122, became the first miRNA-targeted drug tested in humans, initially developed for chronic hepatitis C infection. Clinical studies demonstrated dose-dependent and sustained miR-122 inhibition with an acceptable safety profile. Notably, treated subjects exhibited durable reductions in serum cholesterol, confirming that therapeutic miRNA modulation can yield clinically meaningful lipid effects in humans [187].

This clinical experience provided foundational validation for the miRNA therapeutic platform—demonstrating feasibility, durability, and manageable safety—and directly informed subsequent efforts to apply miRNA modulation to cardiometabolic disease.

### 6.5. Rationale for a Next-Generation Therapeutic Paradigm

The preceding examples—**miR-33, miR-144, and miR-122**—collectively establish three foundational principles for miRNA-based lipid therapeutics that directly inform the rationale for miR-30c [47,50,51] and its liver-directed analog C2 [188]. First, they demonstrate that post-transcriptional network regulation is sufficient to generate clinically meaningful lipid phenotypes, even without direct manipulation of the LDL receptor. Second, translational and clinical experience—most notably antisense inhibition of miR-33 in nonhuman primates and miR-122 in humans—has validated the durability, specificity, and regulatory feasibility of miRNA modulation in cardiometabolic contexts. Third, these canonical miRNAs highlight an important limitation: they primarily influence cholesterol efflux or synthesis but do not directly suppress apoB-containing lipoprotein production, the dominant driver of LDL-C in LDLR-deficient states.

It is precisely within this mechanistic gap—where LDLR-dependent clearance is ineffective and residual LDL-C remains high despite maximal therapy—that miR-30c emerges as mechanistically distinct and clinically compelling. Unlike miR-33, miR-144, or miR-122, miR-30c directly targets microsomal triglyceride transfer protein (MTTP) and related lipid-assembly pathways, thereby reducing hepatic VLDL and apoB secretion at its source in an LDLR-independent manner [47,50,51].

### 6.6. Human Proof-of-Concept for miRNA-Mediated LDL-C Regulation: The miRNA–LDLR Axis

#### Post-Transcriptional miRNA Control of LDL Receptor Expression

LDLR is the dominant determinant of circulating LDL-C, a conclusion supported by decades of human genetics, epidemiology, and interventional studies [189,190,191]. Loss-of-function mutations in LDLR cause familial hypercholesterolemia, whereas increased LDLR activity—whether genetic or pharmacologic—consistently lowers LDL-C and ASCVD risk.

While transcriptional regulation of LDLR via SREBP2 has been extensively characterized, LDLR expression is also strongly regulated post-transcriptionally, a feature that has gained increasing attention in recent years. A defining structural characteristic of LDLR mRNA is its unusually long (~2.5 kb) 3′UTR, which harbors multiple conserved binding sites for miRNAs and RNA-binding proteins [49,192,193]. This architecture makes LDLR particularly sensitive to fine-tuning by noncoding regulatory mechanisms.

Among the miRNAs targeting LDLR, miR-148a emerged as a key post-transcriptional repressor. Functional studies demonstrated that miR-148a binds conserved sites within the LDLR 3′UTR, leading to reduced LDLR protein expression and diminished LDL uptake in hepatocytes [48,49,191]. Importantly, mutation or deletion of these miR-148a binding sites abolishes repression, resulting in increased LDLR expression and enhanced LDL clearance [48,49,193]. In vivo, antisense inhibition of miR-148a increases hepatic LDLR levels and produces significant reductions in plasma LDL-C, confirming physiological relevance beyond cell-based systems [48,49]. Compelling **human genetic validation** of the miRNA–LDLR axis comes from the identification of a rare ~2.5 kb deletion in the distal LDLR 3′UTR (“del2.5”) in Icelandic kindred [48,49]. This deletion shortens the LDLR 3′UTR and removes multiple predicted and experimentally supported miRNA-binding sites, including those recognized by miR-148a. Strikingly, carriers of the del2.5 variant exhibit lifelong lower LDL-C levels and a reduced risk of ASCVD, despite having an intact LDLR coding sequence [48]. Indeed, efforts are being directed to create in vivo deletion of this 3′ UTR of LDLR gene in mice to further strengthen proof-of-concept [193].

These findings established that LDLR abundance is not fixed by coding sequence alone but is dynamically modulated by miRNA-mediated post-transcriptional control—a concept central to understanding polygenic and treatment-resistant hypercholesterolemia.

### 6.7. LDLR-Dependent and LDLR-Independent miRNA Pathways

The miR-148a–LDLR axis exemplifies an LDLR-dependent mechanism of LDL-C lowering through enhanced lipoprotein clearance (Table 6). However, LDLR-dependent strategies have intrinsic limitations in patients with severe HeFH and especially HoFH, where LDLR function is absent or severely impaired [18,165,194,195].

In contrast, miR-30c represents a fundamentally distinct, LDLR-independent miRNA pathway. Rather than increasing LDL clearance, miR-30c lowers circulating atherogenic lipoproteins by suppressing hepatic lipoprotein production at its source. miR-30c directly represses MTTP, a critical factor required for apoB lipidation and VLDL assembly, thereby reducing secretion of apoB-containing lipoproteins [196]. In parallel, miR-30c suppresses de novo lipogenesis and enhances fatty-acid oxidation, preserving hepatic lipid balance and avoiding the steatosis historically associated with direct MTTP inhibition. In addition, miR-30c overexpression lowers LDL-C and significantly attenuates atherosclerosis in LDLR-deficient and ApoE-deficient mouse models [50,197].

## 7. miR-30c as LDLR-Independent LDL-Lowering Modality

### 7.1. miR-30c-Mediated Cholesterol Control

miRNA biology reveals that cholesterol homeostasis is governed not only by coding genes but also by noncoding regulatory circuits that calibrate lipid metabolism over a lifetime. Human genetics (the LDLR 3′UTR deletion) and preclinical intervention (miR-30c modulation) together demonstrate that relief from miRNA-mediated repression can produce durable, cardioprotective lipid phenotypes. Within this framework, miR-30c emerges as a next-generation miRNA therapeutic candidate distinguished by its ability to suppress atherogenic lipoprotein production independently of LDLR function.

Unlike canonical cholesterol miRNAs such as miR-148a, which lower LDL-C by enhancing LDL clearance through increased LDLR abundance, miR-30c targets the hepatic production arm of cholesterol metabolism. This distinction is central to its clinical relevance. In severe hypercholesterolemia—particularly homozygous familial HoFH and subsets of severe HeFH–LDLR-dependent therapies are intrinsically limited because receptor function is absent or severely impaired. miR-30c directly addresses this unmet need by acting upstream of LDL clearance, at the level of lipoprotein assembly and secretion.

### 7.2. Preclinical Evidence of miR-30c Proof-of-Concept

#### 7.2.1. Loss-of-Function Studies

Inhibition or knockdown of miR-30c in hyperlipidemic mouse models (including ApoE^−^/^−^ and LDLR^−^/^−^ backgrounds) resulted in increased MTTP activity, elevated ApoB secretion, worsened hyperlipidemia, and accelerated atherosclerosis, confirming that endogenous miR-30c restrains atherogenic lipoprotein production [50]. Additional loss-of-function studies in vascular and progenitor cell systems revealed context-dependent roles of miR-30c in inflammation, vascular remodeling, and differentiation, underscoring its broader biological footprint beyond lipid metabolism [198,199,200,201].

#### 7.2.2. Gain-of-Function Studies

Conversely, hepatic overexpression or administration of miR-30c mimics consistently produced reductions in MTTP activity, ApoB/VLDL secretion, plasma LDL-C, and atherosclerotic plaque burden across multiple hyperlipidemic mouse models, including ApoE^−^/^−^ and LDLR^−^/^−^ backgrounds [50]. Importantly, these effects were achieved without evidence of hepatic lipid accumulation, reinforcing the mechanistic advantage of miR-30c over direct MTTP inhibition.

Beyond lipid biology, miR-30c overexpression has been shown to exert anti-fibrotic and anti-proliferative effects in cardiac, endothelial, and vascular progenitor contexts through modulation of TGF-β-dependent pathways, highlighting favorable pleiotropic profiles relevant to cardiovascular disease progression [50,51,201,202,203].

#### 7.2.3. miR-30c Differs Fundamentally from Direct MTTP Inhibitors

**Figure 4:**
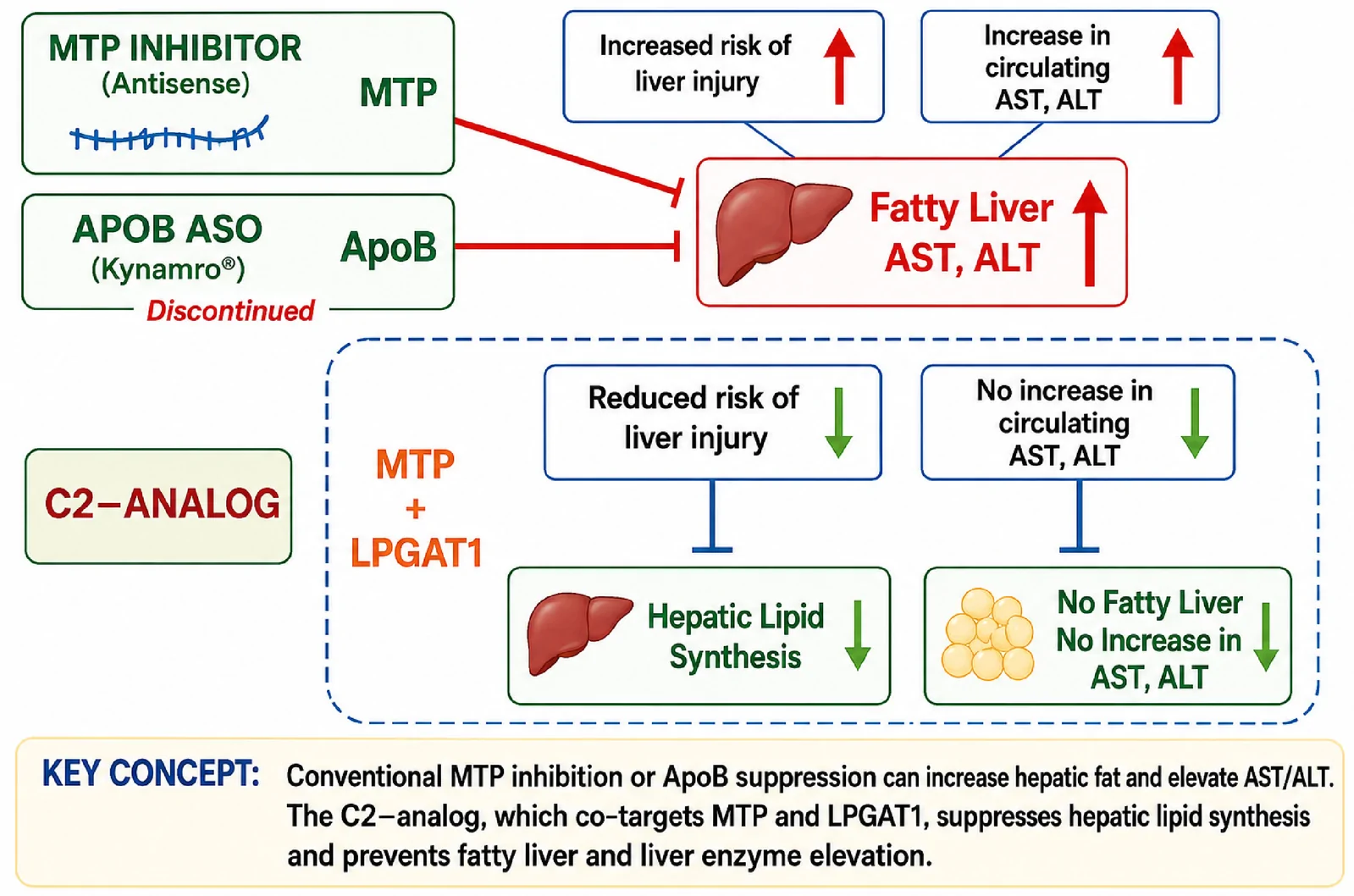
Schematic illustration highlighting the advantages of miR-30c-mediated MTTP inhibition over currently marketed therapies targeting MTTP activity or apoB synthesis. Hepatic assembly and secretion of apoB-containing lipoproteins (VLDL) require two essential components: apolipoprotein B (apoB) and MTTP. Inhibition of MTTP by the small-molecule inhibitor lomitapide (Juxtapid^®^) or suppression of apoB synthesis by the antisense oligonucleotide mipomersen (Kynamro^®^) impairs VLDL assembly and secretion, thereby reducing circulating LDL-C levels. However, because hepatic fatty acid synthesis continues despite impaired lipoprotein export, triglycerides accumulate within hepatocytes, leading to hepatic steatosis (fatty liver) accompanied by elevated plasma AST and ALT levels, reflecting liver injury. In contrast, although miR-30c inhibits MTTP and reduces apoB-containing lipoprotein secretion, it simultaneously suppresses hepatic fatty acid synthesis pathways, including targets such as LPGAT, thereby maintaining hepatic lipid homeostasis. As a result, no significant hepatic fat accumulation or elevations in AST and ALT have been observed in multiple preclinical models treated with miR-30c or its analog C2. **Abbreviations:** MTP, microsomal triglyceride transfer protein; LPGAT, lysophosphatidylglycerol acyltransferase; AST, aspartate aminotransferase; ALT, alanine aminotransferase.

MTTP is essential for the lipidation of apolipoprotein B (apoB) and the assembly of very-low-density lipoprotein (VLDL) particles. By repressing MTTP, miR-30c reduces hepatic VLDL and apoB secretion at its source, thereby lowering downstream LDL-C levels [50,51] (Figure 4). The therapeutic rationale for targeting MTTP is long-standing, as pharmacologic MTTP inhibition can lower apoB-containing lipoproteins. However, MTTP inhibitors were limited by hepatic triglyceride accumulation and steatosis (Figure 4), reflecting uncoupling of lipoprotein secretion from lipid synthesis [204,205]. miR-30c avoids this liability through coordinated pathway regulation. By simultaneously reducing lipoprotein assembly (via MTTP repression) and lipid synthesis—and by promoting lipid utilization—miR-30c preserves hepatic lipid balance in preclinical models (Figure 4). This feature directly reflects the advantage of miRNA-based network calibration and provides a mechanistic explanation for the favorable hepatic tolerability observed in miR-30c overexpression and mimic studies.

#### 7.2.4. miR-30c as a Next-Generation Lipid Therapeutic

**Figure 5:**
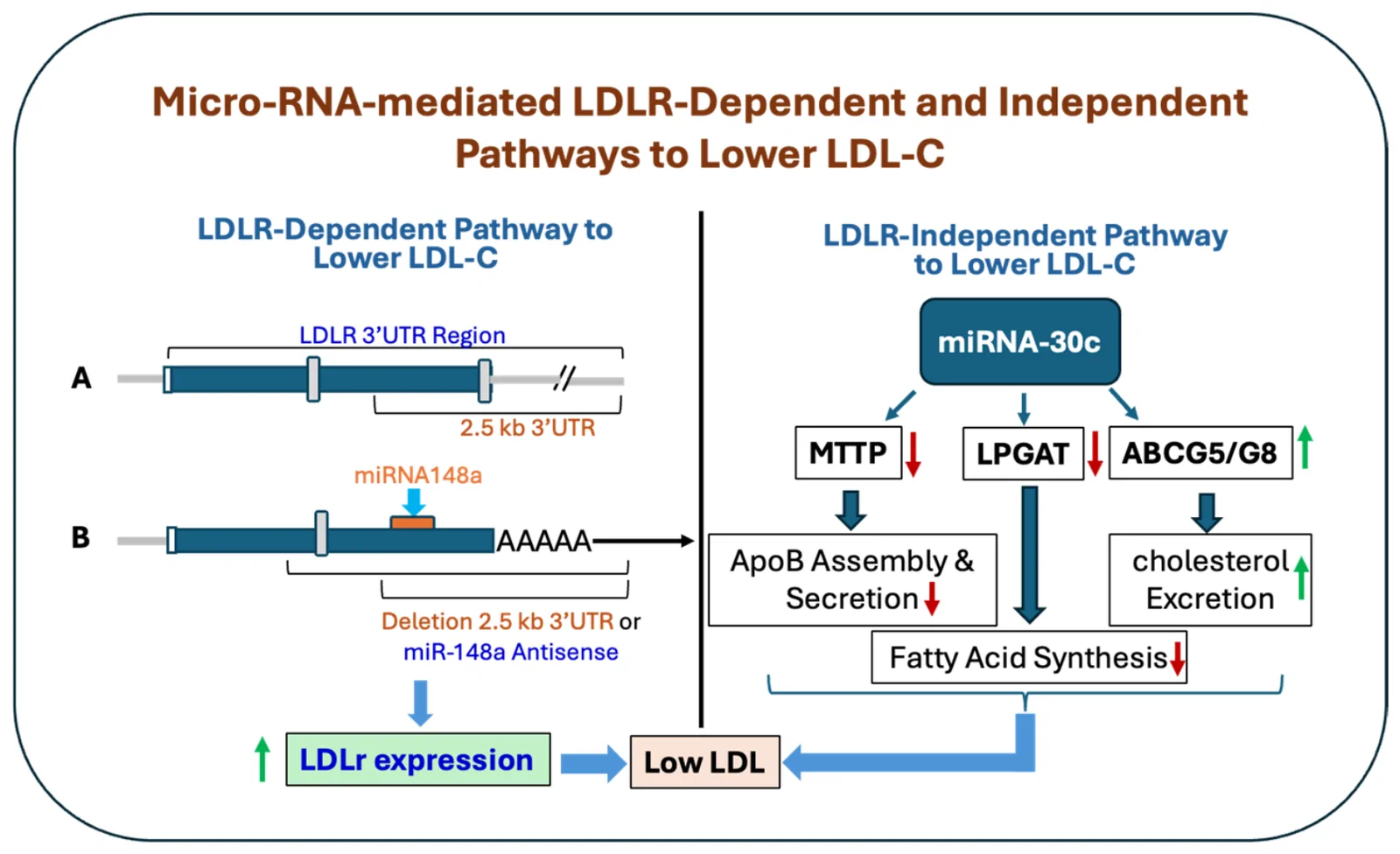
Complementary miRNA control nodes in cholesterol metabolism: miR-148a versus miR-30c. Schematic comparison of LDLR-dependent and LDLR-independent miRNA pathways regulating plasma LDL-C. (**Left**): miR-148a represses LDL receptor (LDLR) expression via binding to the LDLR 3′UTR, reducing hepatic LDL uptake. Relief from miRNA-mediated repression—illustrated by antisense inhibition or the Icelandic LDLR 3′UTR del2.5 variant—enhances LDLR abundance and promotes LDL clearance, primarily benefiting polygenic hypercholesterolemia. (**Right**): miR-30c acts independently of LDLR by repressing microsomal triglyceride transfer protein (MTTP), thereby reducing apoB lipidation and VLDL assembly. Concurrent suppression of de novo lipogenesis by inhibiting fatty acid synthesis and enhancement of fatty-acid oxidation preserves hepatic lipid balance, relevant for LDLR-independent pathway. Together, these pathways illustrate how miRNAs function as network calibrators to achieve durable cholesterol lowering through complementary mechanisms.

Taken together, the progression from human genetic validation of miRNA–LDLR regulation to LDLR-independent miRNA modulation of lipoprotein production illustrates the maturity and understanding of miRNA biology in cholesterol metabolism. miR-30c provides an example of how noncoding regulatory circuits can be therapeutically harnessed to recalibrate lipid flux, rather than simply inhibit single enzymes or receptors (Figure 5).

In this sense, miR-30c/C2 represents not merely another lipid-lowering agent, but a conceptually distinct therapeutic class—one that complements statins, PCSK9 inhibitors, gene therapy, and genome editing, and that may address the residual risk in patients who remain underserved by existing approaches. However, special attention should be placed on the prior observations indicating safety issues in clinical trials, especially immunogenicity and multi-pathway targeting of miRNAs [44,52,53].

### 7.3. Challenges with the Use of miRNAs as Therapeutics

Despite strong biological rationale described above, the clinical translation of miRNA therapeutics has been limited. Only a small number of candidates have entered clinical trials, with none advancing to Phase III; several have been discontinued due to safety concerns [53,206,207]. Notably, most of these clinical efforts have been concentrated in oncology, where the complexity of disease biology further amplifies both opportunity and risk.

A major setback has been toxicity, particularly immune-mediated adverse events. For example, RG-101, a GalNAc-conjugated antagomir targeting miR-122 for HCV, showed early promise but its trial (NCT01829971) was terminated following severe immune reactions [208,209,210]. This highlights the safety risks associated with systemic modulation of miRNAs, especially when inhibitory approaches are employed.

A fundamental challenge lies in the pleiotropic nature of miRNAs. A single miRNA can regulate up to hundreds of mRNAs across multiple pathways [211], while individual genes are often controlled by multiple miRNAs [212]. Although the effect on any single target may be modest [176], the cumulative impact can significantly alter cellular networks. Importantly, inhibition of miRNAs—whether through antagomirs or small molecules—can simultaneously suppress or activate multiple biological pathways, increasing the risk of unintended consequences and safety liabilities. In contrast, miRNA mimics, when delivered in a tissue-specific manner, are less likely to produce such widespread dysregulation, offering a potentially safer therapeutic strategy.

Efficient and specific delivery remains another major barrier. miRNAs are inherently unstable and require precise targeting to specific tissues while avoiding off-target distribution. Although advances such as lipid nanoparticles and GalNAc conjugation have improved delivery, achieving consistent, safe, and tissue-selective delivery continues to be a critical challenge.

Additional concerns include immunogenicity, toxicity, and limitations in sensitivity, specificity, and selectivity. In oncology, miRNAs also contribute to immune evasion and chemotherapy resistance, often driven by tumor microenvironmental factors such as hypoxia [213], further complicating therapeutic intervention.

Given these challenges, careful design and selection of miRNA-based strategies are essential. With these considerations in mind, further studies are being conducted to achieve efficacy without observable adverse effects across multiple preclinical models, including non-human primates [214,215,216].

The concerns mentioned above have tempered early enthusiasm for miRNA drugs and underscore the need for carefully engineered context-specific approaches. In this regard, miR-30c analog C2 illustrates how rational molecular design and liver-focused development by biologists and chemists [188], in preclinical models, address some of the historical limitations of the miRNA modality, but detailed safety and clinical development path still remain challenging.

#### 7.3.1. Pharmacokinetics, Biodistribution, and Safety: Directing miRNA Activity to the Liver

A central challenge in miRNA therapeutics is **precise tissue targeting**. Because miRNAs can regulate multiple transcripts across diverse tissues, unintended distribution may lead to off-target biological effects, particularly in organs such as the brain, immune system, or reproductive tissues.

**Figure 6:**
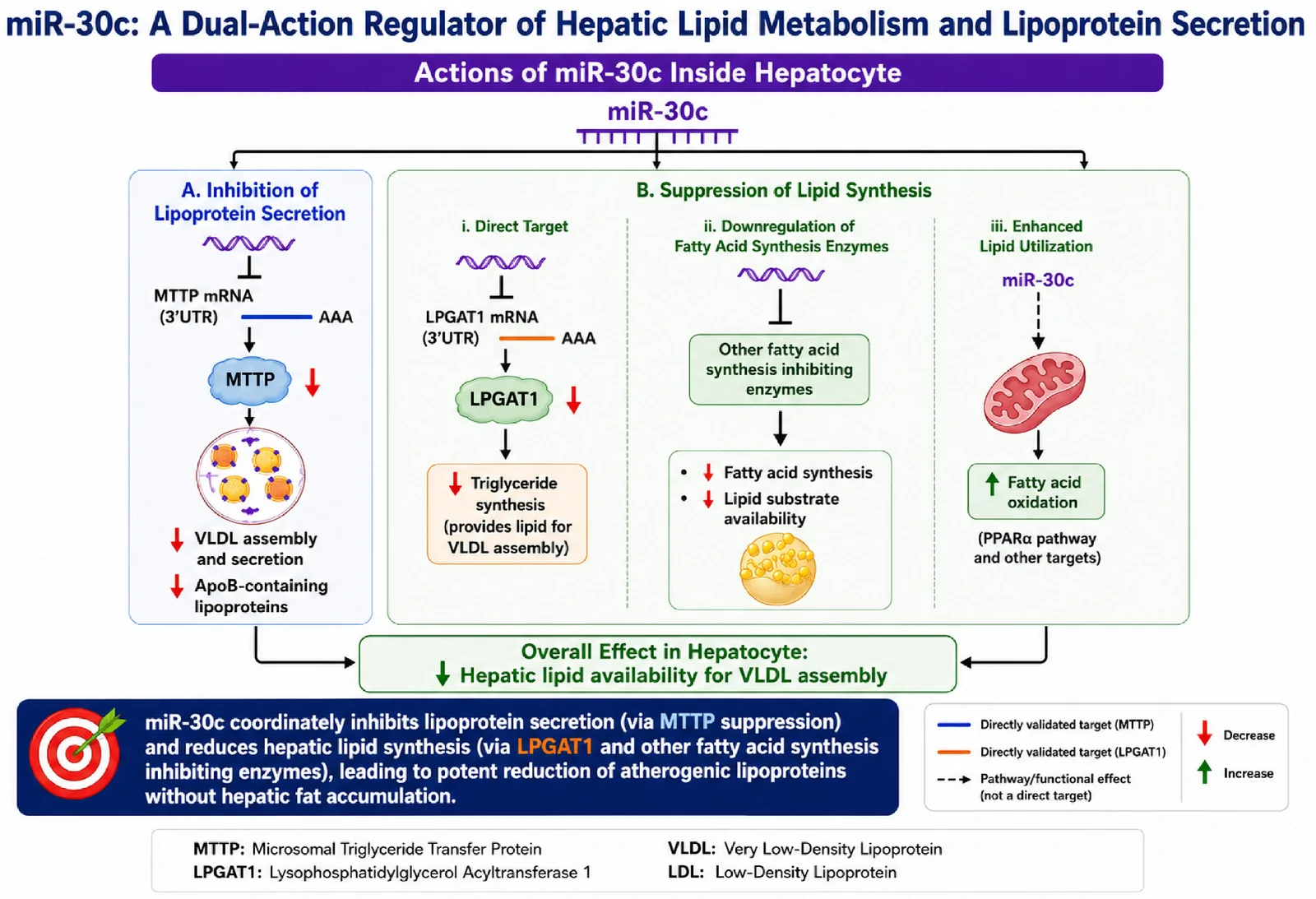
miRNA30c coordinately inhibits lipoprotein secretion via MTTP suppression and reduced hepatic lipid synthesis via inhibition of LPGAT and other fatty acid synthesis enzymes. In addition, miRNA30c also enhances fatty acid oxidation and degradation which contributes to the reduction in the hepatic lipid content. Together, these coordinated actions of miR30c in the liver leads to potent reduction of atherogenic lipoproteins in the blood without hepatic fat accumulation.

Preclinical pharmacokinetic and biodistribution studies of miR-30c analog C2 in mice [51,188] and non-human primates demonstrate that C2 accumulates predominantly in the liver, with minimal or undetectable levels in extrahepatic tissues, including brain, spleen, and kidney. These findings confirm that C2 achieves functional compartmentalization of miR-30c activity, restricting its biological effects to hepatocytes—the primary site of lipoprotein production and cholesterol metabolism (Figure 6). In addition, miR30c showed a dose–response relationship without observable side effects related to liver lipid accumulation because of MTTP inhibition [188].

Another major barrier to miRNA therapeutics has been either miRNA- or delivery-related immunogenicity. Viral vectors and LNP formulations—while effective for nucleic acid delivery—can trigger innate immune activation, complement responses, and infusion-related reactions, complicating chronic or preventive indications.

In contrast, miR-30c analog C2 does not require viral vectors, LNPs, or complex delivery vehicles [188]. The drug product is administered as a chemically modified oligonucleotide with intrinsic liver tropism, eliminating the need for formulation components that are frequently associated with immunogenicity. This “vehicle-free” design substantially reduces the risk of immune activation attributable to delivery systems, an important differentiator from many siRNA and gene-editing platforms. However, the poor pharmacokinetic properties of miRNA30c analog C2 remain a concern.

#### 7.3.2. Immunogenicity, Inflammatory Signaling and Hepatic Safety with miR-30c Analog C2

Immunogenicity remains one of the most frequently cited concerns in miRNA drug development, particularly following early clinical experiences in oncology where immune-mediated adverse events were observed with certain miRNA mimics.

**Figure 7:**
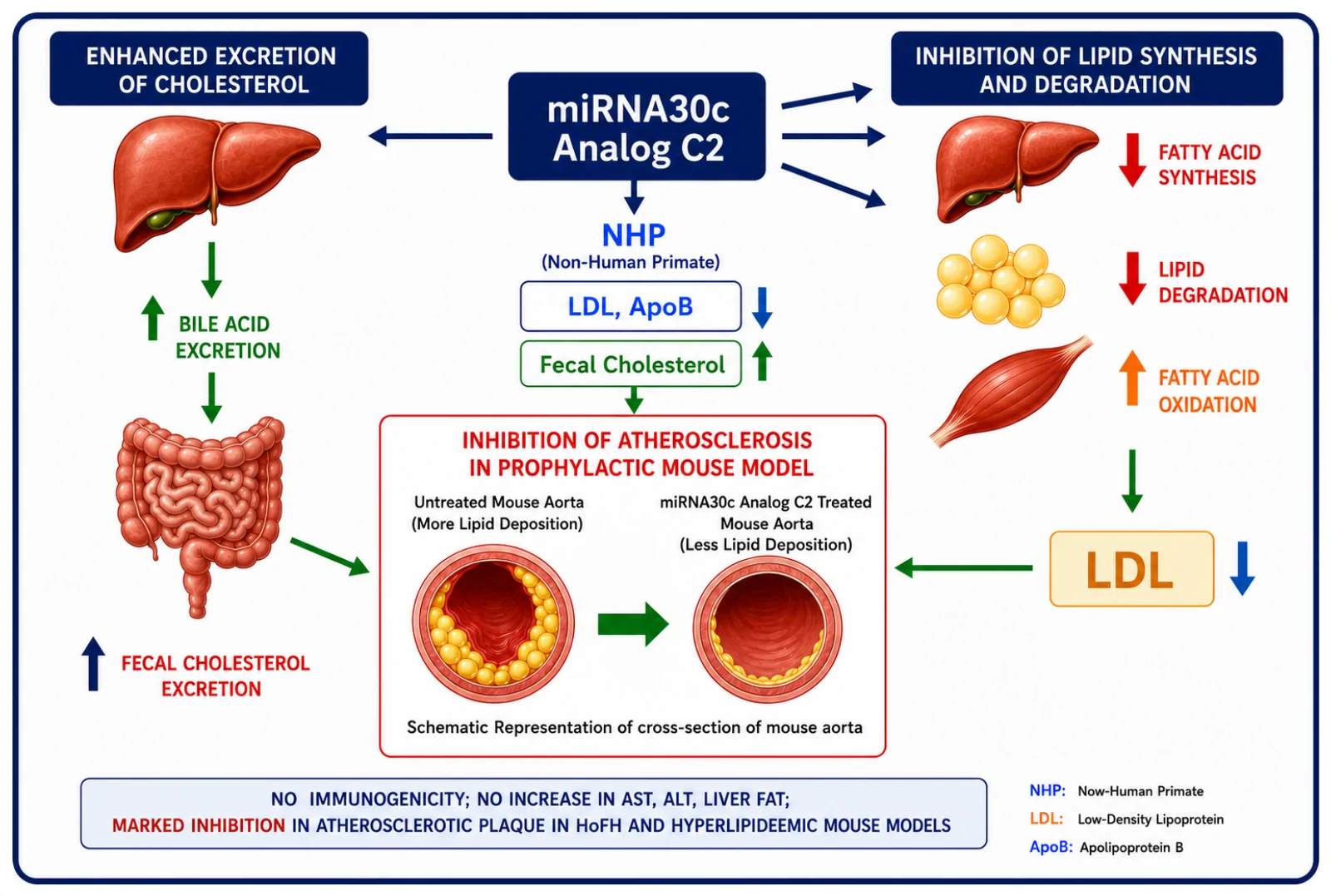
Mode of action of miR-30c analog C2 in inhibiting progression of atherosclerotic plaque. miR-30c analog C2 maintains hepatic lipid homeostasis through a coordinated multi-target mechanism. Its primary action involves downregulation of microsomal triglyceride transfer protein (MTTP), resulting in reduced assembly and secretion of apoB-containing lipoproteins. Simultaneously, miR-30c analog C2 suppresses hepatic fatty acid synthesis through inhibition of lipogenic pathways, including downregulation of LPGAT1. In addition, RNA-seq analyses (216) demonstrated upregulation of genes involved in fatty acid oxidation and lipid degradation. Together, these integrated actions reduce substrate availability for VLDL production while preventing hepatic lipid accumulation. These effects were consistently observed in both mouse models and non-human primates. Beyond its effects on hepatic lipoprotein metabolism, miR-30c analog C2 also enhances whole-body cholesterol elimination by inducing the cholesterol transporters ABCG5 and ABCG8, thereby increasing fecal sterol excretion in both rodents and non-human primates. Collectively, these mechanisms lead to marked anti-atherosclerotic effects, including significant reductions in aortic lipid deposition and plaque burden in hyperlipidemic and HoFH models.

In the case of miR-30c analog C2, extensive long-term preclinical studies across multiple mouse models and non-human primates have not demonstrated evidence of immune activation [217]. Specifically, repeated administration did not result in increases in pro-inflammatory cytokines, including TNF-α, IL-6, or other markers of innate immune stimulation [217]. No signs of systemic inflammation, complement activation, or cytokine release were detected in these studies, supporting a favorable immunological safety profile (Figure 7) [217].

Agents such as mipomersen and small-molecule MTTP inhibitors [204,218] produced accumulation of hepatic triglycerides in animal models and in humans, often accompanied by elevations in AST and ALT, reflecting hepatocellular stress and injury [57]. miR-30c analog C2 differs fundamentally from these approaches. Rather than selectively blocking MTTP activity, miR-30c exerts coordinated regulation of lipid metabolism, simultaneously reducing lipoprotein assembly, suppressing de novo lipogenesis, and enhancing fatty-acid oxidation. This integrated metabolic reprogramming preserves hepatic lipid balance.

### 7.4. Mode of Action of miR30c and Its Analog C2

miR-30c analog C2 follows a replacement (augmentation) strategy, restoring or modestly enhancing the activity of an endogenously protective miRNA rather than shutting down miRNA function entirely. This distinction is critical. By reinforcing a naturally occurring regulatory program that restrains lipoprotein production and lipid synthesis, miR-30c analog C2 achieves metabolic benefit while preserving the broader integrity of cellular regulatory networks. This network-calibrating effect is consistent with the conceptual framework developed, in which miRNAs function as rheostats rather than binary switches.

A central mechanism of miR30c involves direct interaction with the 3′ untranslated region (3′UTR) of MTTP mRNA [50], which is essential for the assembly and secretion of apoB-containing lipoproteins, including VLDL, the precursor of circulating LDL-C. Suppression of MTTP expression by miR-30c or C2 reduces hepatic VLDL secretion, thereby lowering plasma LDL-C levels. Pharmacologic inhibition of MTTP using small molecules [204] or antisense oligonucleotides such as mipomersen [218] has demonstrated LDL-C-lowering efficacy in FH patients but is associated with hepatic steatosis [57] (Figure 4).

In addition to targeting MTTP, miR-30c simultaneously downregulates multiple genes involved in de novo lipogenesis (Figure 4), thereby limiting hepatic fatty acid synthesis. Among these, LPGAT has emerged as a key mediator [51,188]. Furthermore, miR-30c enhances fatty acid β-oxidation pathways in the liver [197], contributing to increased lipid utilization. The coordinated suppression of lipid synthesis and enhancement of lipid catabolism effectively counterbalances reduced lipoprotein secretion, maintaining hepatic lipid homeostasis (Figure 6). Collectively, these integrated actions enable miR-30c and C2 to reduce the production of apoB-containing lipoproteins while preventing hepatic lipid accumulation.

In murine models of HoFH [47] and diet-induced hyperlipidemia [51,217], treatment with miR30c analog C2 resulted in marked reductions in aortic lipid deposition. Further mechanistic studies identified an additional pathway contributing to this effect: induction of the cholesterol transporters ATP-binding cassette transporters G5 and G8 (ABCG5 and ABCG8) (Figure 7), which promote biliary cholesterol excretion [217]. This enhanced reverse cholesterol flux represents a complementary mechanism facilitating whole-body cholesterol clearance and contributing to reduced arterial lipid burden.

A common concern in translational research is the potential disconnect between rodent models and human physiology. Importantly, the efficacy and mechanistic profile of miR-30c have been validated in non-human primates (Figure 7) [217], models considered highly predictive of human lipid metabolism. These studies confirmed consistent reductions in plasma lipids without evidence of hepatic steatosis, supporting the translational robustness of this approach. The high evolutionary conservation of miR-30c sequence across species further reinforces the likelihood of similar biological effects in humans, but as emphasized in this article multiple times, a careful long-term animal safety study followed by a human study are set criteria by regulatory agencies for a new chemical entity or new modality to gain validation.

## 8. Durable Lipid Lowering: Four Emerging Paradigms

The therapeutic landscape for severe hypercholesterolemia is undergoing a structural transition from chronic pharmacologic inhibition toward durable, pathway-level interventions. Three mechanistically distinct paradigms now coexist:

1. **Gene therapy**, aimed at restoring or augmenting gene function (e.g., LDLR replacement);
2. **Genome editing**, designed to permanently silence or modify lipid genes (e.g., PCSK9, ANGPTL3) (Table 7);
3. **siRNA therapy,** designed to guide the selective degradation of complementary messenger RNAs to silence causative genes (Table 7);
4. **miRNA network modulation**, exemplified by miR-30c/C2, which recalibrates lipid flux through coordinated post-transcriptional regulation Table 7).

Each paradigm addresses a different biological leverage point, carries distinct safety and regulatory implications, and may be best suited to different patient populations and risk tolerances [123,159,168,219].

**Table 7:** Positioning of preclinical stage C2 Analog with other treatment modalities, available or in clinical development stage, for HoFH and Difficult to Treat Patients.

Therapies	Mechanism	Patients	Advantages	Disadvantages	Clinical Stage
**PCSK-9**	LDL-R	High LDL	Combined with Statins	LDL-R-dependent, some patients are non-responsive, does not work in HoFH patients [36,37,38]	siRNA Therapy Approved
**Angptl3**	Lipolysis	High TG	Combined with Statins	LDL-R-independent, LDL-lowering mechanism for HoFH not well understood, side effects reported [78,79]	Clinical Development Stage
**Kynamro/Juxtrapid**	LDL production	High LDL, FH patients	None	LDL-R-independent, causes fatty liver and adverse effects, Kynamro discontinued [57,59,62]	Antisense Therapy Approved in HoFH
**miRNA-30c C2 Analog**	LDL Production	High LDL, FH patients	Combined with other therapies in pre-clinical studies, no safety concern in preclinical study [220]	LDL-R-independent. Preclinical stage [197,217]. Long-term studies warranted. More stable product needed for therapeutic development	New Modality in Preclinical Stage

### 8.1. Gene Therapy: Strengths and Structural Limitations

AAV-mediated LDLR gene therapy represents a rational approach for HoFH driven by absent or severely defective receptor function. Preclinical studies in LDLR-deficient mice [94] and WHHL rabbits [84,92] demonstrated sustained LDL-C lowering and attenuation of atherosclerosis, supporting translational feasibility. These data enabled first-in-human trials of liver-directed AAV-LDLR gene transfer in adults with HoFH [114].

However, gene therapy faces intrinsic constraints. AAV vectors are limited by packaging capacity [96], pre-existing anti-capsid immunity [97], and restricted redosing feasibility, while long-term durability and immunologic consequences require extended surveillance [85,98,113,115]. Importantly, gene therapy efficacy depends on functional transgene expression, which may vary with vector dose, hepatocyte turnover, and immune responses. These considerations confine LDLR gene therapy to small, high-risk populations where benefit clearly outweighs long-term uncertainty.

### 8.2. Genome Editing: Promise and Permanence

Genome editing offers the theoretical advantage of one-time, permanent modification of lipid pathways. In vivo base editing of PCSK9 in non-human primates achieved durable LDL-C reductions after a single administration, representing a major milestone for cardiometabolic editing [135,136]. Early clinical translation of PCSK9 editing programs (e.g., VERVE-101 and VERVE-102) signals the field’s momentum [140,143]. Parallel efforts targeting ANGPTL3 provide LDLR-independent lipid lowering relevant to FH biology [142] (Table 7).

Yet, genome editing introduces unique ethical, regulatory, and safety challenges. Permanent genomic alteration raises concerns regarding off-target editing, bystander mutations, irreversibility, and long-term oncogenic risk, necessitating unusually stringent nonclinical packages and long-term follow-up commitments [123,159,168]. For a prevalent, largely preventive condition such as hypercholesterolemia, regulators and clinicians must carefully balance population-level benefit against low-probability but high-impact risks.

### 8.3. siRNA Technology: A Balance of Dosing Frequency and Efficacy

The clinical success of siRNA therapeutics has established them as a major milestone in nucleic acid-based medicine, offering potent and durable gene silencing with infrequent dosing intervals that improve patient convenience and adherence. However, despite these advantages, siRNA therapy has inherent limitations. Gene silencing is transient rather than permanent, necessitating lifelong repeat dosing to maintain therapeutic efficacy. In addition, the clinical benefit of siRNA therapies remains dependent on the integrity of downstream biological pathways. For example, siRNA-mediated inhibition of PCSK9 requires functional LDL receptors to achieve LDL-C lowering, thereby limiting its effectiveness in patients with HoFH who have little or no residual LDL receptor activity. Moreover, most siRNA therapeutics are designed to silence a single gene, an approach that may be insufficient for complex, polygenic metabolic disorders involving multiple interconnected pathways. Nevertheless, the remarkable clinical and regulatory success of siRNA has set a high benchmark for nucleic acid therapeutics and has substantially de-risked the development of next-generation platforms (Table 7). These include antisense oligonucleotides, miRNA mimics, gene therapies, and genome-editing approaches, each addressing distinct therapeutic needs within lipid management.

### 8.4. miRNA Technology: A Middle Ground Between Reversibility and Durability

miRNAs occupy a strategic middle ground between chronic pharmacotherapy and irreversible genomic intervention. By acting at the RNA regulatory layer, miRNA modulates lipid metabolism durably but reversibly [47,50], offering sustained biological effect without permanent DNA modification.

Critically, miR-30c analog C2 targets the production side of the cholesterol balance equation. By repressing MTTP and coordinating suppression of de novo lipogenesis while enhancing fatty-acid oxidation, miR-30c reduces apoB-containing lipoprotein output at its hepatic source [47,50,221].

Despite its promising preclinical efficacy, the current miR-30c analog C2 has pharmacokinetic limitations that necessitate once-weekly intravenous administration to maintain therapeutic activity in animal models. Such a dosing regimen is unlikely to be practical for the chronic treatment of hypercholesterolemia. Therefore, further optimization through chemical modifications and formulation strategies will be required to improve metabolic stability, prolong tissue exposure, and enable subcutaneous administration at longer dosing intervals, ideally once monthly, while preserving efficacy and safety (Table 7). These optimization efforts are important step toward clinical translation. Ultimately, carefully designed clinical studies will be required to determine whether an optimized miR-30c analog demonstrates an acceptable safety profile, durable pharmacokinetic properties, and sustained lipid-lowering efficacy in humans comparable to those observed in preclinical models. Until such clinical evidence becomes available, the therapeutic potential of miR-30c analogs should be interpreted with appropriate caution.

### 8.5. Safety, Reversibility, and Regulatory Alignment

A comparative positioning of the major nucleic acid-based therapeutic platforms is presented in Table 8. Antisense oligonucleotides and siRNA therapeutics have demonstrated clinical efficacy with durable LDL-C lowering and infrequent dosing, whereas gene therapy and genome editing offer the potential for long-lasting, and possibly curative, treatment of inherited dyslipidemias. In contrast, miRNA-based therapeutics regulate multiple genes within interconnected metabolic pathways and may provide broader metabolic benefits. While challenges were encountered in developing miRNA-based therapies [208,209,222], the liver-directed miR-30c analog C2 has shown favorable preclinical characteristics, including liver-restricted biodistribution, vehicle-free delivery, and the absence of detectable innate immune activation or cytokine induction in long-term studies [217]. An important advantage of antisense, siRNA, and miRNA therapeutics is that their pharmacological effects are reversible, allowing dose adjustment or discontinuation if unexpected adverse effects occur—a flexibility that is not available with genome editing and is only partially achievable with gene therapy. This reversibility may be particularly advantageous for preventive indications and for treatment of broader patient populations. However, in the case of miRNA30c analog C2, pharmacokinetic limitation remains a challenge that needs further refinement.

### 8.6. Concluding Perspective

The remarkable progress achieved in nucleic acid therapeutics over the past decade has fundamentally transformed the treatment landscape for hypercholesterolemia. Antisense oligonucleotides and siRNA therapeutics have provided compelling clinical proof-of-concept that selective hepatic gene silencing can produce robust, durable, and infrequently dosed reductions in atherogenic lipoproteins. Gene replacement and in vivo genome-editing technologies offer the prospect of long-lasting and potentially curative interventions for inherited disorders such as familial hypercholesterolemia, although their long-term safety, durability, accessibility, and ethical considerations remain to be fully established.

MicroRNA-based therapeutics represent another emerging class of nucleic acid medicines with a distinct biological mechanism. Unlike antisense oligonucleotides or siRNAs, which generally target a single transcript, miRNAs regulate networks of genes involved in interconnected metabolic pathways. This systems-level regulation has the potential to restore physiological homeostasis but also presents important challenges, including ensuring target specificity, minimizing unintended effects on non-target genes, and optimizing tissue-selective delivery.

Among the miRNA candidates under investigation, the liver-directed miR-30c analog C2 has shown encouraging efficacy in multiple preclinical models through coordinated regulation of hepatic lipoprotein secretion, lipid synthesis, and cholesterol elimination. However, these findings are limited to preclinical studies, and no miRNA-based therapeutic has yet been approved for hypercholesterolemia or any other disease. Before clinical evaluation, significant additional work is needed to optimize pharmacokinetic and pharmacodynamic properties, improve delivery and dosing, establish long-term safety and biodistribution, evaluate potential off-target effects, and complete IND-enabling toxicology and manufacturing studies. Ultimately, its clinical utility will need to be demonstrated in well-designed human trials.

Continued advances in RNA chemistry, targeted delivery technologies, and genome engineering are expected to further improve the safety, efficacy, and clinical applicability of nucleic acid-based therapeutics. Together, these complementary platforms are expanding the therapeutic landscape and hold considerable promise for advancing precision medicine in dyslipidemia and cardiovascular disease.

## Figures and Tables

**Table 1:** Limitations of Statins in U.S. Hyperlipidemia Management [21,31,32].

Unmet Issue	% of Treated Patients	U.S. Patients (Est.)
Intolerance (partial or complete)	7–9%	3.7–4.8 million
Inadequate LDL-C response/resistance	15–20%	8–11 million
Any treatment-related side effects	20–30%	10–16 million
Discontinuation or poor adherence	20–25%	10–13 million

Statin Landscape (Standard of Care). ~53 million U.S. adults treated with statins. First-line therapy across primary & secondary cardiovascular prevention.

**Table 2:** Limitations of PCSK9 Monoclonal Antibodies in U.S. Hyperlipidemia Management [36,37,38] Current PCSK9 mAb Landscape (Alirocumab, Evolocumab).

Unmet Issue	% of Treated Patients	U.S. Patients (Est.)
Inadequate LDL-C response/resistance	5–8%	55,000–90,000
Intolerance leading to discontinuation	3–5%	33,000–55,000
Any treatment-related side effects	10–15%	110,000–165,000
Serious adverse events	<1%	<10,000

~1.1–1.3 million U.S. patients treated to date. Primarily ASCVD, HeFH, and statin-intolerant populations.

**Table 3:** Key milestones in RNA-based lipid therapeutics.

Approx. Year	Modality	Target	Lead Agent	Key Achievement	Key Limitation
1998–2002	Early antisense	Various	First-gen ASOs	Demonstrated feasibility of mRNA targeting	Poor stability, toxicity
2005–2010	2nd-gen antisense	APOB	Mipomersen	First LDL-C lowering via hepatic mRNA silencing in humans	Hepatic steatosis, weekly dosing
2012–2015	RNAi (preclinical)	PCSK9	Multiple	Potent catalytic mRNA cleavage	Delivery challenges
2016–2020	RNAi (clinical)	PCSK9	Inclisiran	Durable LDL-C lowering with twice-yearly dosing	LDLR-dependent
2020–2023	RNAi expansion	LP(a)	Olpasiran	First profound Lp(a) lowering	Outcomes pending
2024–present	Network RNA	Multiple	miRNA programs	Coordinated metabolic regulation	Requires precise targeting

**Table 5:** Chronological milestones in genome editing relevant to hypercholesterolemia.

Year	Milestone	What Was Demonstrated	Relevance
2021	In vivo PCSK9 base editing in nonhuman primates	Durable PCSK9 and LDL-C reduction after one dose	Key preclinical proof-of-concept [135,136]
2021	First systemic in vivo CRISPR editing in humans (ATTR)	Feasible LNP liver delivery; durable effect	Field-level validation [137]
2022–present	First-in-human PCSK9 editing	Clinical translation initiated	Entry into cardiometabolic trials [138,139,140]
2023	ANGPTL3 editing with optimized GalNAc-LNP	LDLR-independent lipid lowering	Relevance to FH biology [141,142]
2023–present	Next-gen PCSK9 editing (VERVE-102)	Platform optimization	Improved efficiency/tolerability [141,143]
2025	Expanded human systemic editing data (ATTR)	Durable benefit; safety insights	Reinforces feasibility [144]
2023–2026	Regulatory harmonization (ICH S12, FDA/EMA guidance)	Clearer BD, CMC, LTFU expectations	Shapes IND/CTA strategy

**Table 6:** Complementary miRNA control nodes in cholesterol metabolism.

Pathway	Primary Mechanism	LDLR Dependence	Clinical Relevance
**miR-148a**	Represses LDL clearance via LDLR downregulation	LDLR-dependent	Polygenic hypercholesterolemia
**miR-30c**	Suppresses hepatic lipoprotein production (MTTP)	LDLR-independent	HoFH, severe HeFH, treatment resistance

**Table 8:** Comparative positioning across therapeutic classes for the treatment of hypercholesterolemia.

Modality	Mechanism	Durability	Reversibility	LDLR Dependence	Key Limitations
Statins/PCSK9 mAbs	Enzymatic inhibition/clearance	Short-acting	Fully reversible	LDLR-dependent	Residual risk, intolerance [21,31,32]
Antisense oligonucleotides (ASOs)	RNase H-mediated degradation of target mRNA	Weeks to months	Yes	Target-dependent	Injection-site reactions, thrombocytopenia (agent-specific), repeated dosing
siRNA therapeutics	RISC-mediated degradation of target mRNA	Months	Yes	Target-dependent	Repeated dosing, delivery restricted primarily to liver
Gene therapy (AAV-LDLR)	Gene replacement	Long-term	Limited	LDLR-dependent	Vector immunity, limited re-dosing, manufacturing complexity [114]
Genome editing	Permanent gene silencing	Permanent	None	Target-dependent	Irreversibility, off-target risk, long-term safety
miR-30c/C2	Network modulation of lipoprotein production	Durable	Yes	LDLR-independent	Clinical translation, long-term efficacy and safety yet to be established

## Data Availability

No new data were created or analyzed in this study. Data sharing is not applicable to this article.

## Abbreviations

AAV	adeno-associated virus
ABE	adenosine base editors
ABCA1/ABCG1	ATP-binding cassette transporters
ANGPTL3	Angiopoietin-like protein 3
ASO	Antisense oligo
ASCVD	atherosclerotic cardiovascular disease
ASGPR	asialoglycoprotein receptor
AST	aspartate aminotransferase
ALT	alanine aminotransferase
CAD	coronary artery disease
CHD	coronary heart disease
Cas-9	CRISPR-associated proteins
CRISPR	clustered regularly interspaced short palindromic repeats
DSBs	double-strand breaks
dsRNA	Double-stranded RNA
FH/FHBL	familial hypobetalipoproteinemia
FXR	Farnesoid X receptor
GalNAC	N-acetylgalactosamine
GLP	Good laboratory practices
HeFH	heterozygous familial hypercholesterolemia
HoFH	homozygous familial hypercholesterolemia
LDL	low-density lipoprotein cholesterol
LDLR	low-density lipoprotein receptor
LNP	lipid nanoparticle
Lp(a)	lipoprotein(a)
LPGAT	Lysophosphatidylglycerol Acyltransferase
miRNA	microRNA
MTTP/MTP	microsomal triglyceride transfer protein
NHEJ	non-homologous end joining
NHP	Non-human primate
PCSK9	proprotein convertase subtilisin/kexin type 9
pegRNA	prime editing guide RNA
pri-miRNA	primary microRNA
RH	refractory hypercholesterolemia
RISC	RNA-induced silencing complex
siRNA	small interfering RNAs
SREBP	sterol regulatory element-binding protein
LCAT	LCAT, lecithin:cholesterol acyltransferase
LNP	lipid nanoparticles
LPL	lipoprotein lipase
mAB	monoclonal antibody
TRL	triglyceride-rich lipoproteins
UTR	Untranslated region
WHHL	Watanabe heritable hyperlipidemic
WT	wild type
